# Comprehensive Empirical Model of Substitution—Influence on Hydrogen Bonding in Aromatic Schiff Bases

**DOI:** 10.3390/ijms232012439

**Published:** 2022-10-18

**Authors:** Katarzyna M. Krupka, Michał Pocheć, Jarosław J. Panek, Aneta Jezierska

**Affiliations:** Faculty of Chemistry, University of Wrocław, ul. F. Joliot-Curie 14, 50-383 Wrocław, Poland

**Keywords:** density functional theory, polarizable continnum model, quantum theory of atoms in molecules, non-covalent interactions, hydrogen bond, proton transfer, schiff base, salen-type ligand

## Abstract

In this work, over 500 structures of tri-ring aromatic Schiff bases with different substitution patterns were investigated to develop a unified description of the substituent effect on the intramolecular hydrogen bridge. Both proximal and distal effects were examined using Density Functional Theory (DFT) in the gas phase and with solvent reaction field (Polarizable Continuum Model (PCM) and water as the solvent). In order to investigate and characterize the non-covalent interactions, a topological analysis was performed using the Quantum Theory of Atoms In Molecules (QTAIM) theory and Non-Covalent Interactions (NCI) index. The obtained results were summarized as the generalized, empirical model of the composite substituent effect, assessed using an additional group of simple ring-based Schiff bases. The composite substituent effect has been divided into separate increments describing the different interactions of the hydrogen bridge and the substituent: the classical substituent effect, involving resonance and induction mediated through the ring, steric increment based on substituent proximity to the bridge elements, and distal increment, derived from substitution on the distal ring.

## 1. Introduction

Substitution is one of the most important aspects of modern chemical science [1,2,3]. It can have different levels of practical impact—from modulating properties of a molecule to providing additional functionalities or reaction paths. Historically, it has been described using the Hammett and σ constants [4,5], which are sometimes used today. Later many new descriptors were introduced, e.g., charge of the Substituent Active Region (cSAR) [6], Substituent Effect Stabilization Energy (SESE) [7,8], and Harmonic Oscillator Model of Aromaticity (HOMA) [9]. They can be calculated using different parameters of the studied compound—from structural and energy-based formulas to the ones using charge distribution [10]. From the point of view of physical chemistry and mechanistic view of reactivity, some of these concepts belong to the realm of “free energy relationships”, a successful framework vigorously developed and investigated for almost a century [11,12].

One of the most interesting (and potentially intricate) effects stemming from substitution is its influence on the hydrogen bonds (HBs) that may be located at or near the substituted part of the molecule [13]. HB is a non-covalent interaction, occurring when in the neighborhood of an X–H entity (where X is the donor atom), a second entity of the specific type is present (called the acceptor) [14,15]. Hydrogen bonding is an important and diverse molecular feature—it can be classified according to strength [16,17], symmetry (difference in electronegativity of donor and acceptor atoms, special shape of potential energy well) [18], acceptor-donor locations (intra- or intermolecular) [19] or even partially covalent character [20,21]. Hydrogen bonds can influence, e.g., reactivity, macro- and microscopic structure and molecular features, influence coordination in complexes, stabilize structure [21,22,23,24,25,26,27]. In the contemporary science, the concept of hydrogen bonding is constantly evolving [28,29,30,31]. While in the typical case the hydrogen atom is close to the donor atom, the phenomenon of proton transfer is also a common occurrence [32]. It can be characterized by the activation energy and depth of the second minimum, associated with the proton path between the donor and acceptor atoms. This behavior can be studied via classical analytical methods (such as IR or NMR spectroscopy [26,33]), but it has also been proven that computational chemistry provides efficient tools for its description [27,34] by means of static or molecular dynamics simulations [13]. Intramolecular hydrogen bonds are one of the most significant molecular interactions responsible for the structure and properties of compounds under study—Schiff bases.

‘Schiff bases’ is a general term covering a wide group of imines, whose structure is characterized by the presence of a hydrocarbonyl group on the nitrogen atom (R${}_{2}$C = NR’, R’ ≠ H). Following the recommendation of IUPAC, they shall be considered synonymous with azomethines [35]. The overwhelming majority of Schiff bases are artificially obtained in condensation reactions of primary amines with aldehydes or ketones [36,37,38]. However, they could also be found in natural products in which corrin-related compounds are present [39]. The first to synthesize azomethines was Hugo Schiff. In 1864 he described the chemistry of ’a new series of organic bases’, which years later were named ’Schiff bases’ in his honor [40,41].

Schiff bases find a number of applications in modern organic synthesis, material science, catalysis and biochemistry [42,43,44,45,46,47,48,49]. For this reason they have been a popular area of scientific interest for many years. Azomethines and their derivatives show catalytic properties as oxidation [50,51,52] and reduction [53,54,55] catalysts. Due to their extensive biological functionality, such as antifungal [56], antiviral [57,58], anticancer [59,60] and antibacterial [61,62] activity, they are also commonly used in medicine. It is essential to mention, that Schiff bases act as excellent ligands for the p-, d- and f-block metal ions, and their complexes often show the strongest of these properties, creating innovative application capabilities [63,64,65,66].

In the studied molecules, two azomethine bridges are located in the vicinity of two acidic hydroxyl groups [67,68,69]. Compounds of this type have strong coordination properties and perform well as polydentate ligands for transition metals [70,71,72,73]. The best-known representative of compounds with a similar structure is salen [74]. The manganese(III) and salen-type ligand complexes remain well-known for their ability to enantioselectively convert prochiral alkenes to epoxides in the Jacobsen epoxidation [75,76,77,78]. Coordination compounds with other d-block metals are being used as biosensors [79,80,81] and catalysts in asymmetric synthesis [82,83,84]. They also play a key role in advanced drug delivery systems in cancer therapy [85,86,87].

In this work, the first element of smart design and assessment of compounds is proposed. Apart from viability and CPU-to-laboratory transitionability, rational resource management is one of the most crucial aspects of modern design regimens. The model presented can rationalize fundamental aspects of design, allowing for semi-quantitative assessment of compounds, with the follow-up studies building upon this idea—transition from empirical increments to basic molecular parameters and static-to-dynamic transitional models, among others, can further optimize design concepts. The sheer scale of this project allowed for in-depth insight into the effects contributing to the proton transfer phenomenon, generalizing the description to be potential application to a wide range of compounds. In particular, formation of quasi-rings due to the intramolecular HB has profound impact on the molecular properties: structural (distances and conformations; aromaticity; electron density distribution), spectroscopic (vibrational—IR; NMR) and related to energy (conformational preferences; reactivity such as ability of complexation of metal ions). Our motivation is to present a general framework which could be in principle implemented for any of these properties, and describe its detailed application to the parameters of the hydrogen bonding energetic profile. This, on one hand, explains large number of the involved structures necessary to develop a thorough model, on the other hand it is also a source of our decision to compose our model on the basis of the energy parameters only.

## 2. Results

### 2.1. Density Functional Theory (DFT)

First, the analysis of the activation energy, the second energy minimum and the difference between these two parameters was performed for the proton transfer in the hydrogen bridges. The computations were performed for both in the gas phase and with solvent reaction field. The results were grouped by parent compound type and analyzed for the proximal and distal effects of the substitution.

**Figure 1:**
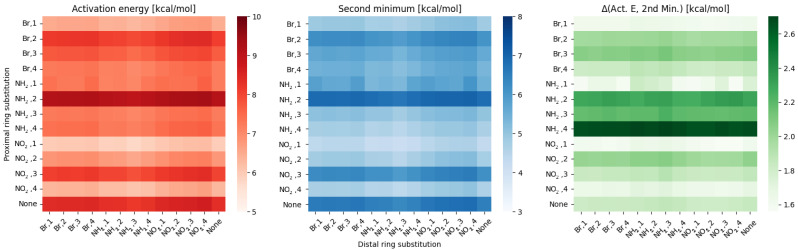
Heatmaps of the activation energy (**left**), second minimum (**middle**) and the difference between them (**right**) for different substitution patterns in the meta compounds group.

For the meta compounds in the gas phase, the results are presented in Figure 1.

From the Figure 1, it is clear that the proximal substitution has a much greater effect on the scanned hydrogen bridge than the distal one. This result is evidenced by distinct changes between each row, while the columns can be grouped as slightly different shades within each substituent. This is also supported by statistics—the standard deviation for the proximal effect is 0.88 ± 0.1, while for the distal effect is 0.14 ± 0.01. All of the data from statistical analysis (mean, standard deviation, extremes) can be found in Supplement B: Selected parameters, Tables SB1–SB6 for the gas phase, and Tables SB7–SB12 for the PCM.

**Figure 2:**
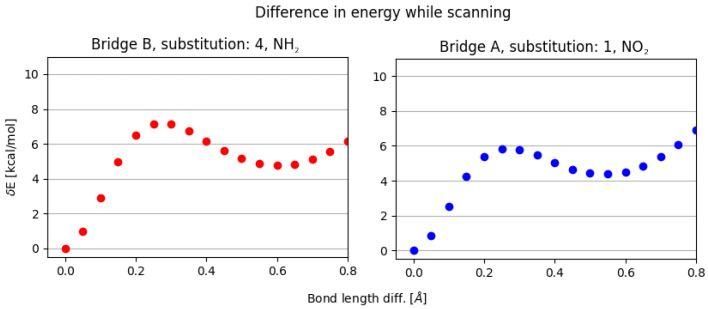
Illustrative energy profiles for scanning of the hydrogen bridge with proximal substitution: NH${}_{2}$, 4 (**left**) and NO${}_{2}$, 1 (**right**).

As for the difference between the activation energy and parameters of the second minimum—it indicates how ‘flat’ the energy profile will be. The largest difference is visible for the **NH${}_{2}$, 4** substitution on proximal ring, while the lowest for **NO${}_{2}$, 1** (**NH${}_{2}$, 1** following as the second closest). This corresponds well with the energy profiles depicted in Figure 2.

**Figure 3:**
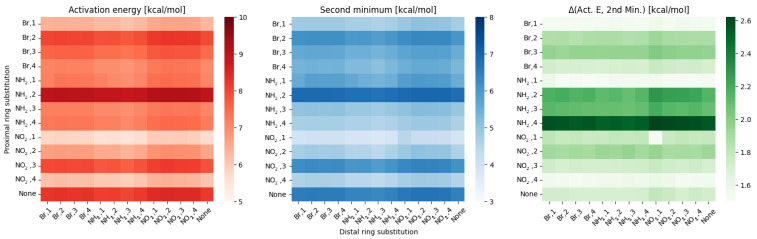
Heatmaps of the activation energy (**left**), second minimum (**middle**) and the difference between them (**right**) for different substitution patterns in the para compounds group.

Some similarities to the meta group are visible within substitutions in the para group, depicted in Figure 3.

While the activation energies for the proximal substitution retain the same pattern and variance, the distal effect gives even better defined ‘areas’ for each substitution type. While in the meta group the position of the distal substitution within the substituent type had a slight, but noticeable impact, in para the effects are even smaller, with the dominating factor being the distal substituent type. The statistical analysis yields similar results as in the meta group—standard deviation for the proximal effect is 0.89 ± 0.1, while for the distal effect is 0.15 ± 0.01.

The difference between the bridge parameters also gives a similar picture as in the meta, with the only major change being **NH${}_{2}$, 1** replacing **NO${}_{2}$, 1** as the substitution pattern.

**Figure 4:**
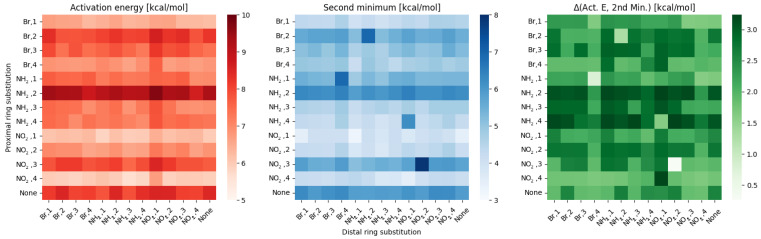
Heatmaps of the activation energy (**left**), second minimum (**middle**) and the difference between them (**right**) for different substitution patterns in the ortho compounds group.

More changes are visible within the last group, ortho, as depicted in Figure 4.

Here, the variance between rows remains significant, but even within a single row the variance is bigger when compared to meta and para groups. This is reflected not only in disappearing of the distal substitution ‘zones’, but also in the statistical descriptors—standard deviation for proximal effect is 0.92 ± 0.1, while for the distal effect significantly raises to around 0.24 ± 0.08. The proximal–distal effects in the hydrogen bridge parameters for all of the groups will be further investigated in Discussion.

Results for the PCM using water as the solvent mostly follow a similar pattern as in the gas phase. For para and meta groups, all of the parameters describing the hydrogen bridge are lower by about 5% when compared to the gas phase. The ortho group is, once again, the outlier—the results are almost identical in both models. This is most likely another instance where the strong correlation between bridges is affecting the energy profiles of the hydrogen bridge scans. Such a small influence of the PCM solvent on the potential energy surface was observed in earlier studies on intramolecular hydrogen bonds in naphtho- and anthraquinone derivatives [88,89]. Comparing the energy profiles of the studied compounds in the gas phase and in the solvent, we can see that the presence of solvent does not affect significantly the shape of the energy profile, which is coupled with small impact on the electron density distribution. The PCM counterparts of Figure 1, Figure 3 and Figure 4 can be found in Supplement B: Selected parameters, Figures SB13–SB15.

**Figure 5:**
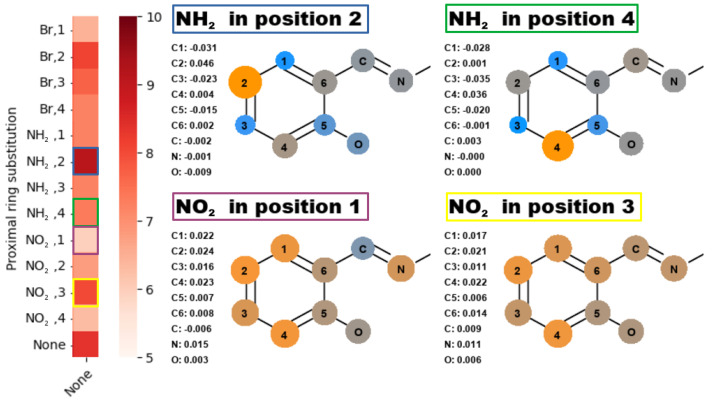
Differences of charge distribution for the selected proximal substitution patterns compared to the non-substituted structure, and related to their respective fields on the activation energy heatmap from Figure 1.

We were studying effects of the substitution on charge distribution within the part of the molecule proximal to the scanned hydrogen bridge. An example of the different distributions for proximal substitution in meta group is depicted in Figure 5, with all of the data in Supplement B: Selected parameters, Figures SB1–SB9.

**Figure 6:**
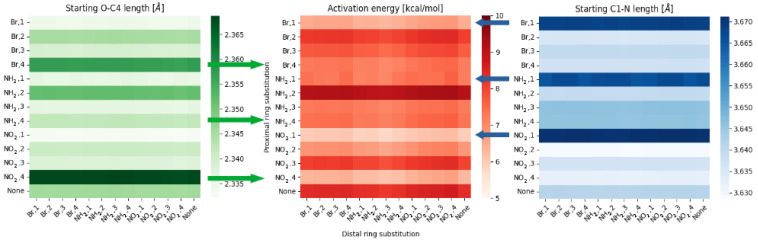
Heatmaps of the activation energy (**middle**), O—C4 distance (**left**) and N—C1 distance (**right**) for different substitution patterns within meta compounds group.

The most meaningful description for the charge distribution in these structures is not using the charges per se, but rather their relation to the unsubstituted counterpart. This gives a very clear indication of substituent effect on electronic structure within the discussed part of the molecule. As indicated on heatmaps from Figure 1, Figure 3 and Figure 4, some irregularities can be found within the substitution patterns, e.g., if **NH${}_{2}$, 2** results in heightened activation energy, **NH${}_{2}$, 4** should reveal a similar effect, but results in lowered activation energy instead, despite almost exact same charge distribution between the two. Similarly, **NO${}_{2}$, 3** has unchanged the activation energy when compared to the base structure, while for **NO${}_{2}$, 1** the activation energy is significantly lowered. To best describe the possible sources of these irregularities, we have performed additional analysis based upon the spatial relation of O and N bridge atoms to the neighboring parts of the analyzed ring. For meta compounds this is depicted in Figure 6, while all of the data can be found in Supplement B: Selected parameters, Figures SB10–SB12.

When analyzing the data from the O—C4 and N—C1 it is important to remember, that many effects will be affecting each other, forming the final description.

### 2.2. Quantum Theory of Atoms in Molecules

To reveal the presence of intramolecular hydrogen bonds, the analysis of the electron density based on Quantum Theory of Atoms In Molecules (QTAIM) formalism [90] was performed. For the investigation, the structures monosubstituted in proximal positions 1 and 4 were chosen, due to the coexistence of classical substituent effect, affecting the bridge via resonance and induction, with additional ‘steric’ effect.

**Figure 7:**
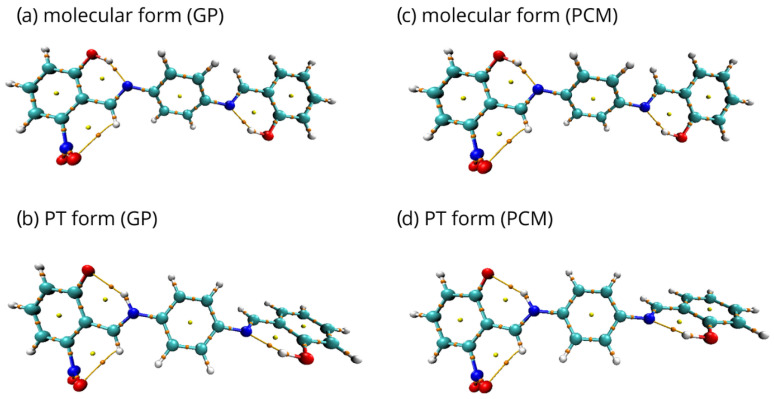
QTAIM topology maps for the para isomer monosubstituted with –NO${}_{2}$ group in the proximal position 1: (**a**) molecular form in the gas phase, (**b**) proton-transferred form in the gas phase, (**c**) molecular form in PCM, (**d**) proton-transferred form in PCM. The orange and yellow dots mark the BCPs and RCPs, respectively. Yellow lines pinpoint the intramolecular interaction paths.

Topology maps are presented in Figure 7 for para isomer monosubstituted with –NO${}_{2}$ group in proximal position 1 and in Supplement C: Topological analysis, Figures SC1–SC7 for the remaining structures. They represent visualizations of electronic structure properties based on the QTAIM description, namely, the critical points (BCPs and RCPs), which are stationary points of the electron density field. Regardless of the type of substituent and the complexity of the effects affecting the electron density, the quasi-ring systems were formed and recognized by the RCPs. The BCPs of covalent bonds and the indicated bond paths of the hydrogen bridges were also recognized. The presence of intramolecular hydrogen bonds significantly stabilizes the conformation of the molecules. In addition, the intramolecular interactions involving –Br and –NO${}_{2}$ groups were detected. The quasi-rings, as characterized by the presence of the BCPs and RCPs, were found, indicating that the C-H...O intramolecular hydrogen bonds and C-H...Br halogen bonds were formed.

The electronic structure analysis was carried out for molecular and proton-transferred (PT) forms. The selected results of the analysis are presented in Table 1 and Table 2 for the para isomer monosubstituted with –NO${}_{2}$ group in the proximal position 1, and in Supplement C: Topological analysis, Tables SC1–SC14 for the other structures. First, net atomic charges in the quasi-rings were calculated. The partial charge value of the donor oxygen atom is lower when it remains bonded to the bridged proton. A decrease in the net charge at the acceptor nitrogen atom is observed for the PT form. It is noteworthy that the hydrogen atoms are more positively charged when they are bonded to the donor oxygen atom. For all of the analyzed compounds, the sum of partial QTAIM charges on the quasi-ring was decreased in the PT form compared to the molecular form. Next, the values of electron density and their Laplacian at Bond Critical Points (BCPs) of intramolecular hydrogen bond in each studied molecule were computed. The electron density Laplacians at BCPs are negative for both: O-H interaction in the molecular forms and N-H in the PT form, indicating covalent bonds. However, the electron density values are lower for the O-H bonds in the molecular forms. This demonstrates, that the O-H bonds are stronger than N-H bonds formed after proton transfer.

Although the PCM solvent environment affects the polarization of the O-H and N-H bonds, the involvement of the atoms in the intramolecular hydrogen bonding and quasi-ring systems brings larger stability to the electron density distribution and values of the partial atomic charges [91,92]. Consequently, the results for the PCM using water as the solvent are analogous to those obtained in the gas phase. This fact of similar electron density distribution in both phases is in agreement with our earlier remarks on the energetic parameters. The PCM results confirmed our expectations, based on previous experience [88,89], that the introduction of solvent did not change the molecular covalent skeleton topology.

### 2.3. Non-Covalent Interactions Index

The Non-Covalent Interactions (NCI) index [93] was implemented to explore intramolecular non-bonded contacts in the selected compounds and make the weak interactions between substituents and hydrogen bridge atoms more specific. The Reduced Density Gradient (RDG) scatter graphs were generated between RDG function and sign($\lambda_{2}$)ρ, where sign($\lambda_{2}$)ρ signifies the electron density multiplied by the sign of the second Hessian eigenvalue ($\lambda_{2}$). The value of sign($\lambda_{2}$)ρ refers to the strength and nature of the weak interactions.

**Figure 8:**
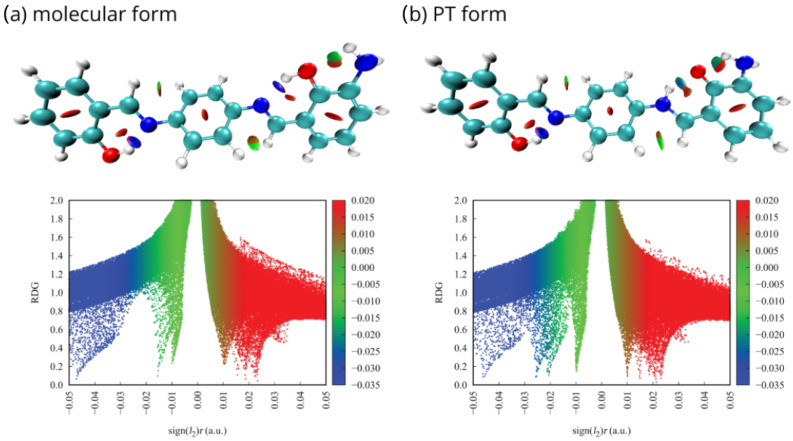
NCI gradient isosurfaces and scatter graphs for the para isomer monosubstituted with the –NH${}_{2}$ group in the proximal position 4 in the gas phase: (**a**) molecular form, (**b**) proton-transferred form.

Sign($\lambda_{2}$)ρ > 0 is the typical value for repulsion, e.g., steric effect in ring, sign ($\lambda_{2}$)ρ≈ 0 for van der Waals interactions and sign ($\lambda_{2}$)ρ < 0 for attraction, such as in hydrogen or halogen bonds. The interactions were depicted as the gradient isosurfaces and scatter graphs, which are presented in Figure 8 for the para isomer monosubstituted with the –NH${}_{2}$ group in the proximal position 4 in the gas phase. Visualizations for the other structures can be found in Supplement C: Topological analysis, Figures SC8–SC21. The red, green and blue regions represent repulsion, van der Waals interaction and strong attraction, respectively.

Application of the NCI visual method allowed to confirm the presence of interactions detected by QTAIM analysis, as well as observe the weak interactions, which were not recognized before. In the molecular forms, the blue isosurfaces between acceptor atom (N) and the bridge proton (H) result from the strong H-bonds (H...N). They could be also observed as the blue regions in the RDG scatter graphs. In the PT form, where the proton is attached to the acceptor atom (N-H), the H-bond (O...H) is significantly weaker and mixed blue-green regions are observed. Red isosurfaces in the centre of the aromatic and quasi-rings refer to repulsive interactions with the delocalized π-electrons in the rings. However, the most significant weak interactions were detected between the –NH${}_{2}$, –NO${}_{2}$ and –Br substituents and atoms involved in the hydrogen bridge or located in its close vicinity. The molecular contacts presented as the green van der Waals regions indicate the presence of substantial (NH${}_{2}$, 4–OH), (NO${}_{2}$, 1–CH), (Br, 4–OH), and (Br, 1–CH) interactions—from the strongest to the weakest. They are responsible for forming additional quasi-rings, affecting the hydrogen bridge and proton transfer phenomena, causing additional ‘steric’ substituent effect discussed earlier. Moreover, the PT contributes to the increase of an attractive nature of aforementioned interactions (presented as mixed green–blue regions).

Implementation of the implicit PCM approach with water as the solvent did not affect the RDG function significantly. The NCI analysis for molecules in PCM follows the pattern of the results obtained in the gas phase, in accordance with generally small impact of the PCM model on the QTAIM properties, noted in the previous section.

## 3. Discussion

One of the most important elements of the results from our studies is the coexistence and cross-influence of two effects coming from the substitution on the ring proximal to the scanned hydrogen bridge:

- classical substituent effect, affecting the hydrogen bridge via resonance and induction effects;
- ‘steric’ effect—some of the substituents can have additional, non-trivial effect while in positions 1 or 4.

Effects coming from the resonance and induction are best assessed using the charge distribution across the proximal ring. When comparing data from Supplement B: Selected parameters, Figures SB1–SB9 it is clear, that (in agreement with the classic description of this effect) the substitution gives the same effects in charge distribution, within error margin, in positional pairs: 1–3 and 2–4. This should result in the same energy profiles for the positions within a given pair, but it is not the case, as mentioned while presenting Figure 5. This inconsistency should be attributed to the ‘steric’ effect.

It is visible in Figure 6, that the substitution at position 1 will have a significant impact on the N–C1 distance, what translates to the rings rotation in the C6 point—this will result in shortening of the O-N bridge distance, lowering the activation energy of the proton transfer. On the other hand, a substitution at position 4 will push the O-H group towards the N atom. Both of these effects can be somewhat counteracted by the charge distribution, additional rotations and deformations, but will have significant consequences for the proton transfer. It is also worth noting that –NH${}_{2}$ can form additional hydrogen bonds if suitable acceptor atom is in range—as in the NH${}_{2}$, 4 pattern. This behavior affects the bridge oxygen—with an additional proton with –NH${}_{2}$ forming a secondary hydrogen bridge, the primary bridged proton’s transfer will have a lower activation energy. This possibility was confirmed using the NCI index.

**Figure 9:**
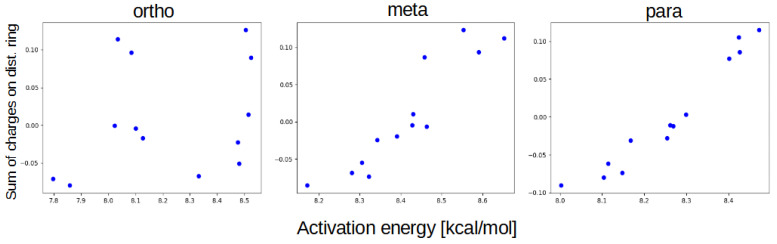
Relationship between the sum of the charges on the distal ring and the activation energy of the proton transfer for different groups of compounds.

When comparing results, it is also clearly visible that the effects of the distal substitution on the scanned hydrogen bridge vary between different groups—from changing almost exclusively between substituent types (Br, NH${}_{2}$, and NO${}_{2}$) in para and meta, to changes more akin to the proximal substitution pattern in ortho. We believe that this is the effect of bridge-bridge correlation—the sterical proximity of the two bridges translates to the significant cross-bridge impact. In para and meta, hydrogen bridges almost do not affect each other, and this results in the potential energy profile being correlated almost exclusively to the summary charges on the distal ring (Figure 9).

In ortho, however, the correlation between bridges results in differences not only in summary charges on the distal part, but mostly due to the second hydrogen bridges energy parameters, as the effects are very similar to the effects of the proximal substitution (but with lower magnitudes).

In the final part of the analysis, we decided to build an empirical model of the ‘composite substituent effect’ $E_{A}$, which can be described by the equation:

(1) $$E_{A}=E_{BC}+I_{E-SUB}+I_{E-STER}+I_{E-DIST}$$

where $E_{BC}$ is the activation energy for the base compound, $I_{E-SUB}$ is the increment from resonance and inductive effect of substitution through the ring (classical substituent effect), $I_{E-STER}$ is the increment from summary effect of substituents proximity to the bridge (steric and electrostatic) and $I_{E-DIST}$ is the increment from the distal part of the compound.

The final part, $I_{E-DIST}$, is only applicable to the entities from this research (or similar), but the generalized version of the equation:

(2) $$E_{A}=E_{BC}+I_{E-SUB}+I_{E-STER}$$

can be used to describe other Schiff bases that have –OH and –C=N– groups at the adjacent positions on the ring.

The model describing empirically derived values for increments $I_{E-SUB}$ and $I_{E-STER}$ for different monosubstitution schemes, based on the analysis of para and meta groups, is presented below as Table 3. These two groups were chosen because the bridged protons are not correlated as in ortho group, what should translate to the better generalization of the description. Calculating $I_{E-SUB}$ we assumed that the substitution at position 1 and 3 for each compound has the same effect on the activation energy, and the same is assumed for positions 2 and 4. This is due to the nature of the classical substituent effect (as described earlier in the discussion of net atomic charge distributions), and can be done also because any possible differences can be accounted for in $I_{E-STER}$ part (but are mostly negligible, as seen for NO${}_{2}$, 1 $I_{E-SUB}$ part). The $I_{E-STER}$ is calculated as a difference between corresponding (1–3 and 4–2) substitution positions.

Same as for the activation energy, the equation for the second minimum of the proton transfer can be generally described as:

(3) $$S_{M}=S_{BC}+I_{S-SUB}+I_{S-STER}$$

with the specific parts corresponding to those from the Equation (2).

**Figure 10:**
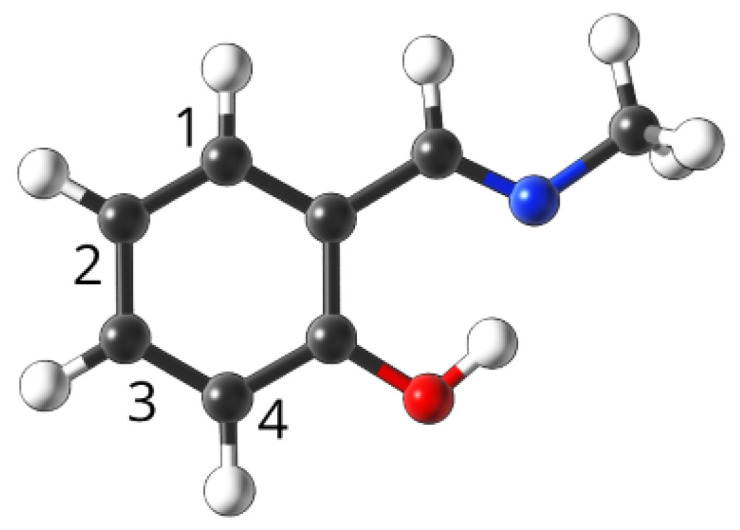
Base compound used to test the empirical model, with substitution sites indicated by numbers.

The model was self-assessed using the whole database, and the approximation error did not exceed 2%. It was also checked using a new group of compounds, with the simplified base structure as shown in Figure 10.

For the mono substituted derivatives, the maximal approximation errors were estimated as 2% for the activation energy and 6% for the second minimum. The higher errors are likely the results of larger conformational flexibility—the –CH${}_{3}$ group, that is, the effective replacement of the remainder of the original molecule, has much lower mass and gives much lower sterical and electrostatic tension when ‘pushed’ by the effects of the substitution.

We also assessed the viability of the Equations (2) and (3) in describing double substituted derivatives. In that case, most of the errors stayed within 1–5%, with some notable exceptions:

1. NO${}_{2}$, 1; NO${}_{2}$, 4—the sterical increments should result in more pronounced lowering of the parameters, but experimental values of the activation energy and the second minimum are higher than expected. We believe, this is the effect of the repulsion between O and N in the hydrogen bridge, acting like a spring—the more it is compressed, the more force is needed to further shorten it.
2. NO${}_{2}$, 1; NH${}_{2}$, 4—the model value is larger than the experimental one, because of the NH${}_{2}$–OH interaction, which can act as dampener for the counteractive effect described above.
3. NO${}_{2}$, 2; NH${}_{2}$, 3 and NH${}_{2}$, 2; NO${}_{2}$, 3—this is the most elaborate effect, as it does not affect the molecules geometry explicitly, but rather modulates both of the substituents by the formation of the additional bridge between them. This will highly impact the effect each one has on the ring via resonance and induction.

**Figure 11:**
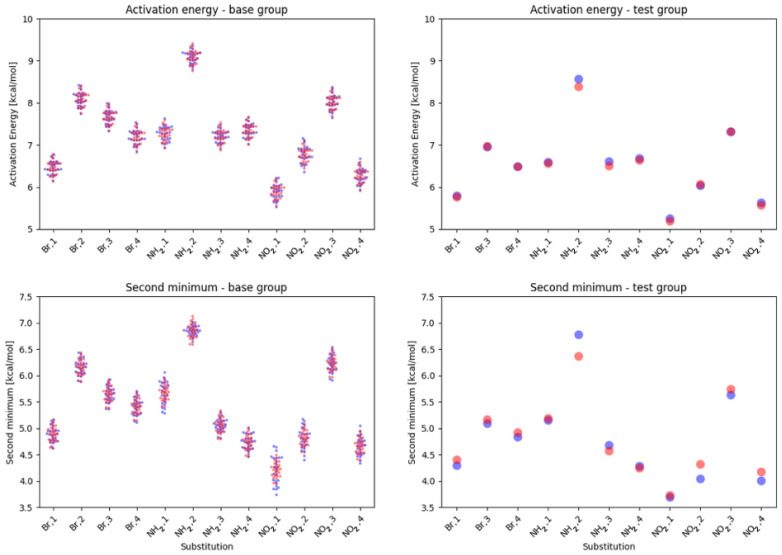
Model performance for base compounds (**left**) and test compounds (**right**). The experimental values are shown in blue, while the model-based values are shown in red.

We believe that the model can be changed from one based on empirical increments to using more basic descriptors such as the number of lone pairs in a substituent, etc., but this warrants further research. The graphical interpretation of the model is presented in Figure 11, while the residuals can be found in Supplement B: Selected parameters, Figure SB16.

We also checked whether this model could be used to evaluate a compound from the group of naphthoquinone [13]. For compounds **2a** (base), **2c** (equivalent of **Br, 4** substitution) and **2b** (equivalent of reversed **Br, 3** substitution) from this paper we applied the model to predict the activation energy and the second minimum of the proton transfer in the compound with not only different basal structure (connected rings), but with the acceptor atom being an oxygen. The model-predicted value of the activation energies were assessed as (**2c**) 11.342 and (**2b**) 13.634 kcal/mol, translating to the errors of 0.1% and 2.9%, respectively. The second minima from the model were calculated to be (**2c**) 10.454 and (**2b**) 10.695 kcal/mol, giving errors of 0.19% and 4.7%. More significant errors (but still within an acceptable range) in the **2b** compound can be explained by the complexity of the molecules core—as the effects from **X, 3** pattern are mostly ring-mediated, the second, conjugated ring acts as a charge ‘sponge’, lowering the resonance and induction effects. This shows, that the model can be used for a wider choice of compounds, but this approach requires further testing.

## 4. Materials and Methods

### 4.1. Studied Compounds

**Figure 12:**
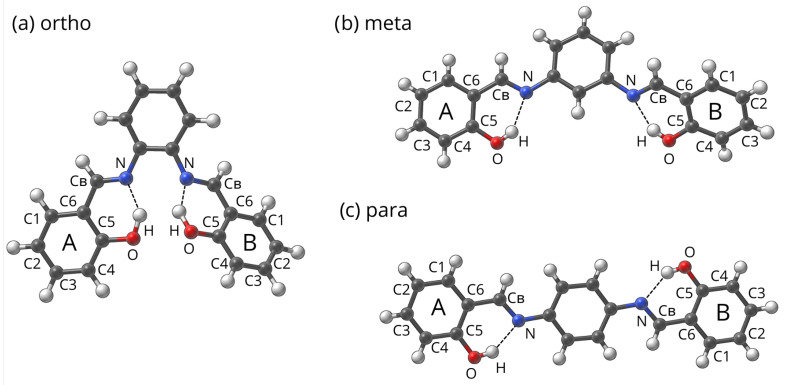
Base structures of studied compounds ((**a**) [95], (**b**) [69], (**c**) [68]).

We have selected 3 parent structures of model Schiff bases from the Cambridge Crystallographic Data Centre (CCDC) [94] named:

- ortho-code 1226150 [95],
- meta-code 828843 [69], and
- para-code 962961 [68],

which are presented in Figure 12.

Names for the groups of compounds in this project were assigned due to the relation on the center ring. In the next step, the substituted derivatives were build using –Br, –NH${}_{2}$ and –NO${}_{2}$, both in single substitution, as well as double substitution (each substituent on separate outer ring) patterns. The substitution patterns were denoted as A, X—B, Y, where A is the type of substituent on ring A, X is the substitution position on ring A, B is the type of substituent on ring B and Y is the substitution position on ring B. The unsubstituted rings are marked as ‘None, None’ for the given ring. Full list of names and substitution patterns is presented in Supplement A: Naming schemes, Tables SA1–SA3.

During the analysis, it was convenient at a certain point to switch from the A-B naming scheme to the proximal–distal (relative to the scanned bridge). The studied base compounds have two symmetrically located HBs, which means if ring B is substituted by –Br on position 3 it was scanned on two bridges—A and B. This gives information on the energy profile of proximal ‘Br, 3’ substitution and distal ‘Br, 3’ substitution. Similarly, with ring A substituted in ‘Br, 3’ pattern and ring B in ‘NO${}_{2}$, 1’ pattern, that means we can acquire data for ‘Proximal Br, 3, Distal NO${}_{2}$, 1’ substitution, but also for ’Proximal NO${}_{2}$, 1, Distal Br, 3’ substitution.

The simple Schiff base for testing was also built from scratch, and then substituted using multiple patterns to evaluate the feasibility of the model. All of the investigated compounds were visualized using the Samson R1 2022 program [96].

### 4.2. Density Functional Theory

The structures of compounds obtained from the CCDC database were investigated using Density Functional Theory (DFT) [97,98]. All computations were performed at the ωB97XD/6-311+G(2d,2p) [99,100,101] level of theory. First, the structures were optimized in both gas phase as well as with continuum solvation model (Polarizable Continuum Model– IEF-PCM formula [102]) using water as the solvent. Structural compatibility with minima on the Potential Energy Surface (PES) was confirmed by analyzing computed harmonic frequencies. Then, the optimized structures were subjected to the hydrogen bond scan—with the O–H–N angle frozen, the O–H distance was elongated by 20 increments of 0.05 Å each. The Hirshfeld atomic charge population was computed, and wavefunction files were prepared for use in topological analysis. The quantum-mechanical simulations were performed using the Gaussian 16 Rev. C.01. suite of programs [103].

Then all the data were subjected to analysis using custom Python scripts, transforming the data and extracting most vital parameters. To alleviate the problems caused by the sheer size of the database, a custom Python package to automate the analysis was prepared [104] and published. The scripts used to build, analyze and model the data are publicly available on GitHub repository [105,106,107]. All scripts use Python ver. 3.10 [108], with packages Pandas 1.4.3 [109], Matplotlib 3.5.3 [110] and Seaborn 0.11.2 [111].

### 4.3. Topological Analysis Methods—Quantum Theory of Atoms in Molecules (QTAIM) and Non-Covalent Interactions (NCI) Index

The topological analysis in the gas phase (GP) and in the PCM was performed for para compounds, monosubstituted with –NH${}_{2}$, –NO${}_{2}$ and –Br on proximal ring in positions 1 and 4. The para group was selected due to the smallest coupling of the hydrogen bridges. Both molecular and proton-transferred (PT) forms were analyzed for each molecule.

The Quantum Theory of Atoms In Molecules (QTAIM) theory [90] was applied to study the electronic structure, topology and proton transfer phenomena in the hydrogen bridge. The parameters describing the Bond and Ring Critical Points (BCPs and RCPs), as well as the partial QTAIM atomic charges for the atoms forming the quasi-ring, were obtained and analyzed. The Non-Covalent Interactions (NCI) index [93] was employed to reveal and investigate areas of weak interactions between ring substituents and other atoms. For this purpose, reduced density gradient (RDG) function was calculated and analyzed.

The QTAIM and NCI calculations were performed using the Multiwfn 3.8 program [112]. The visualizations were prepared using the VMD 1.9.3 [113], Gnuplot 5.4.4 [114] and Adobe Photoshop 23.5 [115] packages.

## 5. Conclusions

In this work, the en masse analysis of the diversely substituted Schiff bases was performed, providing data concerning effects of the substitution pattern and type on the parameters of the hydrogen bridge. It combined the established tools of computational chemistry with statistical analysis using self-made scripts. In addition to the classical ring-mediated substituent effect, it has been found, that the steric proximity of the substitutent to the studied hydrogen bridge can induce additional changes in the activation energy and the second minimum of the proton transfer process, without changing parameters such as charge distribution. It has been also revealed that the substitution of the distal part has an effect on the hydrogen bridge properties, although smaller in magnitude (below 0.5 kcal/mol). All of these effects can be combined as the composite substituent effect. We have proposed a generalized mathematical description for the hydrogen bridge parameters in connection with substitution, based on empirical increments. We tested this model against itself and different test-cases, and obtained approximation errors to a satisfactory extent (below 5%).

## Figures and Tables

**Table 1:** QTAIM Bond Critical Point (BCP) properties for selected bonds in the para isomer monosubstituted with the –NO${}_{2}$ group in the proximal position 1.

	Gas Phase		PCM	
**BCP**	ρBCP [e*$a_{0}^{-3}$]	∇2ρBCP [e*$a_{0}^{-5}$]	ρBCP [e*$a_{0}^{-3}$]	∇2ρBCP [e*$a_{0}^{-5}$]
**Molecular form**				
O-H	0.336	−2.525	0.333	−2.492
H...N	0.056	0.112	0.058	0.112
**Proton-transferred form**				
O...H	0.025	0.082	0.025	0.081
H-N	0.357	−2.206	0.358	−2.210

**Table 2:** QTAIM net atomic charges for atoms forming quasi-ring in the para isomer monosubstituted with the –NO${}_{2}$ group in the proximal position 1.

Net Atomic Charge [e]	Gas Phase	PCM
**Molecular form**		
O	−1.202	−1.187
H	0.664	0.650
N	−0.111	−1.123
C${}_{B}$	0.613	0.651
C6	−0.019	−0.011
C5	0.651	0.634
SUM	−0.404	−0.386
**Proton-transferred form**		
O	−1.146	−1.143
H	0.503	0.505
N	−1.184	−1.184
C${}_{B}$	0.529	0.529
C6	−0.038	−0.041
C5	0.899	0.898
SUM	-0.437	−0.436

**Table 3:** Empirical values of increments for the activation energy and the second minimum in the proton transfer process, calculated on the basis of para and meta groups.

Substitution Pattern	*I*${}_{E-\mathrm{SUB}}$ [kcal/mol]	*I*${}_{S-\mathrm{SUB}}$ [kcal/mol]
Br,1 or Br,3	−0.670	−0.905
Br,2 or Br,4	−0.244	−0.396
NO${}_{2}$, 1 or NO${}_{2}$, 3	−0.322	−1.496
NO${}_{2}$, 2 or NO${}_{2}$, 4	−1.56	0.297
NH${}_{2}$, 1 or NH${}_{2}$, 3	−1.12	−0.324
NH${}_{2}$, 2 or NH${}_{2}$, 4	0.754	−1.747
**Substitution Pattern**	***I*${}_{E-\mathrm{STER}}$ [kcal/mol]**	***I*${}_{S-\mathrm{STER}}$ [kcal/mol]**
Br,1	−1.199	−0.759
Br,4	−0.902	−0.750
NO${}_{2}$, 1	−2.127	0.613
NO${}_{2}$, 4	−0.501	−2.116
NH${}_{2}$, 1	−0.060	−2.016
NH${}_{2}$, 4	−1.743	−0.141

## Data Availability

The data relevant to this article are contained within the article itself and in the Supplementary Material Files.

## Supplementary Materials

The following supporting information can be downloaded at: https://www.mdpi.com/article/10.3390/ijms232012439/s1. Supplement A: Naming scheme, Supplement B: Selected parameters, Supplement C: Topological analysis.

## Abbreviations

The following abbreviations are used in this manuscript:

DFT	Density Functional Theory
PCM	Polarizable Continuum Model
QTAIM	Quantum Theory of Atoms In Molecules
NCI	Non-Covalent Interactions index
HB	Hydrogen Bond
GP	Gas Phase
PT	Proton Transfer
BCP	Bond Critical Point
RCP	Ring Critical Point
RDG	Reduced Density Gradient
CCDC	Cambridge Crystallographic Data Centre
PES	Potential Energy Surface
