# Supplementary material for: Comprehensive Empirical Model of Substitution—Influence on Hydrogen Bonding in Aromatic Schiff Bases
Source: Int J Mol Sci. 2022 Oct 18;23(20):12439. doi: 10.3390/ijms232012439 (PMC9604547; doi:10.3390/ijms232012439)
Supplement: Supplementary file 1 [file ijms-23-12439-s001.zip › Supplement A.pdf]

Supplement SA: Naming scheme

Comprehensive empirical model of substitution – influence on hydrogen bonding in aromatic Schiff bases

*Katarzyna M. Krupka[1], Michał Pocheć[1], Jarosław J. Panek[1] and Aneta Jezierska[1]**

[1]Faculty of Chemistry, University of Wrocław, ul. F. Joliot-Curie 14, 50-383 Wrocław, Poland

Correspondence should be addressed to: aneta.jezierska@chem.uni.wroc.pl.

Table SA1. Ortho naming scheme:

File Name	Type	Ring A sub.	Sub. in position	Ring B sub.	Sub. in position	A sub. ID	B sub. ID
ortobr11	ortho	Br	1	Br	1	Br,1	Br,1
ortobr12	ortho	Br	2	Br	1	Br,2	Br,1
ortobr13	ortho	Br	3	Br	1	Br,3	Br,1
ortobr14	ortho	Br	4	Br	1	Br,4	Br,1
ortobr1	ortho	Br	1	None	None	Br,1	None,None
ortobr22	ortho	Br	2	Br	2	Br,2	Br,2
ortobr23	ortho	Br	3	Br	2	Br,3	Br,2
ortobr24	ortho	Br	4	Br	2	Br,4	Br,2
ortobr2	ortho	Br	2	None	None	Br,2	None,None
ortobr34	ortho	Br	3	Br	4	Br,3	Br,4
ortobr3	ortho	Br	3	None	None	Br,3	None,None
ortobr44	ortho	Br	4	Br	4	Br,4	Br,4
ortobr4	ortho	Br	4	None	None	Br,4	None,None
ortobrnh11	ortho	NH2	1	Br	1	NH2,1	Br,1
ortobrnh12	ortho	NH2	2	Br	1	NH2,2	Br,1
ortobrnh13	ortho	NH2	3	Br	1	NH2,3	Br,1
ortobrnh14	ortho	NH2	4	Br	1	NH2,4	Br,1
ortobrnh22	ortho	NH2	2	Br	2	NH2,2	Br,2
ortobrnh23	ortho	NH2	3	Br	2	NH2,3	Br,2
ortobrnh24	ortho	NH2	4	Br	2	NH2,4	Br,2
ortobrnh33	ortho	NH2	3	Br	3	NH2,3	Br,3
ortobrnh44	ortho	NH2	4	Br	4	NH2,4	Br,4
ortobrno11	ortho	NO2	1	Br	1	NO2,1	Br,1
ortobrno12	ortho	NO2	2	Br	1	NO2,2	Br,1
ortobrno13	ortho	NO2	3	Br	1	NO2,3	Br,1
ortobrno14	ortho	NO2	4	Br	1	NO2,4	Br,1
ortobrno22	ortho	NO2	2	Br	2	NO2,2	Br,2
ortobrno23	ortho	NO2	3	Br	2	NO2,3	Br,2

ortobrno24	ortho	NO2	4	Br	2	NO2,4	Br,2
ortobrno33	ortho	NO2	3	Br	3	NO2,3	Br,3
ortobrno34	ortho	NO2	3	Br	4	NO2,3	Br,4
ortobrno44	ortho	NO2	4	Br	4	NO2,4	Br,4
ortonh11	ortho	NH2	1	NH2	1	NH2,1	NH2,1
ortonh12	ortho	NH2	2	NH2	1	NH2,2	NH2,1
ortonh13	ortho	NH2	3	NH2	1	NH2,3	NH2,1
ortonh14	ortho	NH2	4	NH2	1	NH2,4	NH2,1
ortonh1	ortho	NH2	1	None	None	NH2,1	None,None
ortonh22	ortho	NH2	2	NH2	2	NH2,2	NH2,2
ortonh23	ortho	NH2	3	NH2	2	NH2,3	NH2,2
ortonh24	ortho	NH2	4	NH2	2	NH2,4	NH2,2
ortonh2	ortho	NH2	2	None	None	NH2,2	None,None
ortonh33	ortho	NH2	3	NH2	3	NH2,3	NH2,3
ortonh34	ortho	NH2	3	NH2	4	NH2,3	NH2,4
ortonh3	ortho	NH2	3	None	None	NH2,3	None,None
ortonh44	ortho	NH2	4	NH2	4	NH2,4	NH2,4
ortonh4	ortho	NH2	4	None	None	NH2,4	None,None
ortonhbr12	ortho	Br	2	NH2	1	Br,2	NH2,1
ortonhbr13	ortho	Br	3	NH2	1	Br,3	NH2,1
ortonhbr14	ortho	Br	4	NH2	1	Br,4	NH2,1
ortonhbr23	ortho	Br	3	NH2	2	Br,3	NH2,2
ortonhbr24	ortho	Br	4	NH2	2	Br,4	NH2,2
ortonhbr34	ortho	Br	4	NH2	3	Br,4	NH2,3
ortonhno11	ortho	NO2	1	NH2	1	NO2,1	NH2,1
ortonhno12	ortho	NO2	2	NH2	1	NO2,2	NH2,1
ortonhno13	ortho	NO2	3	NH2	1	NO2,3	NH2,1
ortonhno14	ortho	NO2	4	NH2	1	NO2,4	NH2,1
ortonhno22	ortho	NO2	2	NH2	2	NO2,2	NH2,2
ortonhno23	ortho	NO2	3	NH2	2	NO2,3	NH2,2
ortonhno24	ortho	NO2	4	NH2	2	NO2,4	NH2,2
ortonhno33	ortho	NO2	3	NH2	3	NO2,3	NH2,3
ortonhno34	ortho	NO2	3	NH2	4	NO2,3	NH2,4
ortonhno44	ortho	NO2	4	NH2	4	NO2,4	NH2,4
ortono11	ortho	NO2	1	NO2	1	NO2,1	NO2,1
ortono12	ortho	NO2	2	NO2	1	NO2,2	NO2,1
ortono13	ortho	NO2	3	NO2	1	NO2,3	NO2,1
ortono14	ortho	NO2	4	NO2	1	NO2,4	NO2,1
ortono1	ortho	NO2	1	None	None	NO2,1	None,None
ortono22	ortho	NO2	2	NO2	2	NO2,2	NO2,2
ortono23	ortho	NO2	3	NO2	2	NO2,3	NO2,2
ortono24	ortho	NO2	4	NO2	2	NO2,4	NO2,2
ortono2	ortho	NO2	2	None	None	NO2,2	None,None
ortono33	ortho	NO2	3	NO2	3	NO2,3	NO2,3
ortono34	ortho	NO2	3	NO2	4	NO2,3	NO2,4
ortono3	ortho	NO2	3	None	None	NO2,3	None,None

ortono44	ortho	NO2	4	NO2	4	NO2,4	NO2,4
ortono4	ortho	NO2	4	None	None	NO2,4	None,None
ortonobr12	ortho	Br	2	NO2	1	Br,2	NO2,1
ortonobr13	ortho	Br	3	NO2	1	Br,3	NO2,1
ortonobr14	ortho	Br	4	NO2	1	Br,4	NO2,1
ortonobr23	ortho	Br	3	NO2	2	Br,3	NO2,2
ortonobr24	ortho	Br	4	NO2	2	Br,4	NO2,2
ortonobr34	ortho	Br	4	NO2	3	Br,4	NO2,3
ortononh12	ortho	NH2	2	NO2	1	NH2,2	NO2,1
ortononh13	ortho	NH2	3	NO2	1	NH2,3	NO2,1
ortononh14	ortho	NH2	4	NO2	1	NH2,4	NO2,1
ortononh23	ortho	NH2	3	NO2	2	NH2,3	NO2,2
ortononh24	ortho	NH2	4	NO2	2	NH2,4	NO2,2
ortononh34	ortho	NH2	3	NO2	4	NH2,3	NO2,4
orto	ortho	None	None	None	None	None,None	None,None

Table SA2. Meta naming scheme:

File Name	Type	Ring A sub.	Sub. in position	Ring B sub.	Sub. in position	A sub. ID	B sub. ID
metaBr12	meta	None	None	Br	2	None,None	Br,2
metaBr13	meta	None	None	Br	3	None,None	Br,3
metaBr14	meta	None	None	Br	4	None,None	Br,4
metaBr1	meta	Br	1	None	None	Br,1	None,None
metaBr21	meta	Br	4	Br	1	Br,4	Br,1
metaBr22	meta	Br	1	Br	1	Br,1	Br,1
metaBr23	meta	Br	2	Br	3	Br,2	Br,3
metaBr24	meta	Br	4	Br	4	Br,4	Br,4
metaBr25	meta	Br	3	Br	3	Br,3	Br,3
metaBr26	meta	Br	2	Br	2	Br,2	Br,2
metabr27	meta	Br	1	Br	2	Br,1	Br,2
metabr28	meta	Br	1	Br	3	Br,1	Br,3
metabr29	meta	Br	4	Br	2	Br,4	Br,2
metabr30	meta	Br	4	Br	3	Br,4	Br,3
metabrnh21	meta	Br	4	NH2	1	Br,4	NH2,1
metabrnh22	meta	Br	1	NH2	1	Br,1	NH2,1
metabrnh23	meta	Br	2	NH2	3	Br,2	NH2,3
metabrnh24	meta	Br	4	NH2	4	Br,4	NH2,4
metabrnh25	meta	Br	3	NH2	3	Br,3	NH2,3
metabrnh26	meta	Br	2	NH2	2	Br,2	NH2,2
metabrnh27	meta	Br	1	NH2	2	Br,1	NH2,2
metabrnh28	meta	Br	1	NH2	3	Br,1	NH2,3
metabrnh29	meta	Br	4	NH2	2	Br,4	NH2,2
metabrnh30	meta	Br	4	NH2	3	Br,4	NH2,3
metabrno21	meta	Br	4	NO2	1	Br,4	NO2,1
metabrno22	meta	Br	1	NO2	1	Br,1	NO2,1

metabrno23	meta	Br	2	NO2	3	Br,2	NO2,3
metabrno24	meta	Br	4	NO2	4	Br,4	NO2,4
metabrno25	meta	Br	3	NO2	3	Br,3	NO2,3
metabrno26	meta	Br	2	NO2	2	Br,2	NO2,2
metabrno27	meta	Br	1	NO2	2	Br,1	NO2,2
metabrno28	meta	Br	1	NO2	3	Br,1	NO2,3
metabrno29	meta	Br	4	NO2	2	Br,4	NO2,2
metabrno30	meta	Br	4	NO2	3	Br,4	NO2,3
metanh12	meta	None	None	NH2	2	None,None	NH2,2
metanh13	meta	None	None	NH2	3	None,None	NH2,3
metanh14	meta	None	None	NH2	4	None,None	NH2,4
metanh1	meta	NH2	1	None	None	NH2,1	None,None
metanh21	meta	NH2	4	NH2	1	NH2,4	NH2,1
metanh22	meta	NH2	1	NH2	1	NH2,1	NH2,1
metanh23	meta	NH2	2	NH2	3	NH2,2	NH2,3
metanh24	meta	NH2	4	NH2	4	NH2,4	NH2,4
metanh25	meta	NH2	3	NH2	3	NH2,3	NH2,3
metanh26	meta	NH2	2	NH2	2	NH2,2	NH2,2
metanh27	meta	NH2	1	NH2	2	NH2,1	NH2,2
metanh28	meta	NH2	1	NH2	3	NH2,1	NH2,3
metanh29	meta	NH2	4	NH2	2	NH2,4	NH2,2
metanh30	meta	NH2	4	NH2	3	NH2,4	NH2,3
metanhbr21	meta	NH2	4	Br	1	NH2,4	Br,1
metanhbr23	meta	NH2	2	Br	3	NH2,2	Br,3
metanhbr27	meta	NH2	1	Br	2	NH2,1	Br,2
metanhbr28	meta	NH2	1	Br	3	NH2,1	Br,3
metanhbr29	meta	NH2	4	Br	2	NH2,4	Br,2
metanhbr30	meta	NH2	4	Br	3	NH2,4	Br,3
metanhno21	meta	NO2	4	NH2	1	NO2,4	NH2,1
metanhno22	meta	NO2	1	NH2	1	NO2,1	NH2,1
metanhno23	meta	NO2	2	NH2	3	NO2,2	NH2,3
metanhno24	meta	NO2	4	NH2	4	NO2,4	NH2,4
metanhno25	meta	NO2	3	NH2	3	NO2,3	NH2,3
metanhno26	meta	NO2	2	NH2	2	NO2,2	NH2,2
metanhno27	meta	NO2	1	NH2	2	NO2,1	NH2,2
metanhno28	meta	NO2	1	NH2	3	NO2,1	NH2,3
metanhno29	meta	NO2	4	NH2	2	NO2,4	NH2,2
metanhno30	meta	NO2	4	NH2	3	NO2,4	NH2,3
metano12	meta	None	None	NO2	2	None,None	NO2,2
metano13	meta	None	None	NO2	3	None,None	NO2,3
metano14	meta	None	None	NO2	4	None,None	NO2,4
metano1	meta	NO2	1	None	None	NO2,1	None,None
metano21	meta	NO2	4	NO2	1	NO2,4	NO2,1
metano22	meta	NO2	1	NO2	1	NO2,1	NO2,1
metano23	meta	NO2	2	NO2	3	NO2,2	NO2,3
metano24	meta	NO2	4	NO2	4	NO2,4	NO2,4

metano25	meta	NO2	3	NO2	3	NO2,3	NO2,3
metano26	meta	NO2	2	NO2	2	NO2,2	NO2,2
metano27	meta	NO2	1	NO2	2	NO2,1	NO2,2
metano28	meta	NO2	1	NO2	3	NO2,1	NO2,3
metano29	meta	NO2	4	NO2	2	NO2,4	NO2,2
metano30	meta	NO2	4	NO2	3	NO2,4	NO2,3
metanobr21	meta	NO2	4	Br	1	NO2,4	Br,1
metanobr23	meta	NO2	2	Br	3	NO2,2	Br,3
metanobr27	meta	NO2	1	Br	2	NO2,1	Br,2
metanobr28	meta	NO2	1	Br	3	NO2,1	Br,3
metanobr29	meta	NO2	4	Br	2	NO2,4	Br,2
metanobr30	meta	NO2	4	Br	3	NO2,4	Br,3
metanonh21	meta	NH2	4	NO2	1	NH2,4	NO2,1
metanonh23	meta	NH2	2	NO2	3	NH2,2	NO2,3
metanonh27	meta	NH2	1	NO2	2	NH2,1	NO2,2
metanonh28	meta	NH2	1	NO2	3	NH2,1	NO2,3
metanonh29	meta	NH2	4	NO2	2	NH2,4	NO2,2
metanonh30	meta	NH2	4	NO2	3	NH2,4	NO2,3
meta	meta	None	None	None	None	None,None	None,None

Table SA3. Para naming scheme:

File Name	Type	Ring A sub.	Sub. in position	Ring B sub.	Sub. in position	A sub. ID	B sub. ID
parabr11	para	Br	1	Br	1	Br,1	Br,1
parabr12	para	Br	2	Br	1	Br,2	Br,1
parabr13	para	Br	3	Br	1	Br,3	Br,1
parabr14	para	Br	4	Br	1	Br,4	Br,1
parabr1	para	None	None	Br	1	None,None	Br,1
parabr22	para	Br	2	Br	2	Br,2	Br,2
parabr23	para	Br	3	Br	2	Br,3	Br,2
parabr24	para	Br	4	Br	2	Br,4	Br,2
parabr2	para	None	None	Br	2	None,None	Br,2
parabr33	para	Br	3	Br	3	Br,3	Br,3
parabr34	para	Br	4	Br	3	Br,4	Br,3
parabr3	para	None	None	Br	3	None,None	Br,3
parabr44	para	Br	4	Br	4	Br,4	Br,4
parabr4	para	None	None	Br	4	None,None	Br,4
parabrnh11	para	Br	1	NH2	1	Br,1	NH2,1
parabrnh12	para	Br	1	NH2	2	Br,1	NH2,2
parabrnh13	para	Br	1	NH2	3	Br,1	NH2,3
parabrnh14	para	Br	1	NH2	4	Br,1	NH2,4
parabrnh22	para	Br	2	NH2	2	Br,2	NH2,2
parabrnh23	para	Br	2	NH2	3	Br,2	NH2,3
parabrnh24	para	Br	2	NH2	4	Br,2	NH2,4
parabrnh33	para	Br	3	NH2	3	Br,3	NH2,3

parabrnh34	para	Br	3	NH2	4	Br,3	NH2,4
parabrnh44	para	Br	4	NH2	4	Br,4	NH2,4
parabrno11	para	Br	1	NO2	1	Br,1	NO2,1
parabrno12	para	Br	1	NO2	2	Br,1	NO2,2
parabrno13	para	Br	1	NO2	3	Br,1	NO2,3
parabrno14	para	Br	1	NO2	4	Br,1	NO2,4
parabrno22	para	Br	2	NO2	2	Br,2	NO2,2
parabrno23	para	Br	2	NO2	3	Br,2	NO2,3
parabrno24	para	Br	2	NO2	4	Br,2	NO2,4
parabrno33	para	Br	3	NO2	3	Br,3	NO2,3
parabrno34	para	Br	3	NO2	4	Br,3	NO2,4
parabrno44	para	Br	4	NO2	4	Br,4	NO2,4
paranh11	para	NH2	1	NH2	1	NH2,1	NH2,1
paranh12	para	NH2	2	NH2	1	NH2,2	NH2,1
paranh13	para	NH2	3	NH2	1	NH2,3	NH2,1
paranh14	para	NH2	4	NH2	1	NH2,4	NH2,1
paranh1	para	None	None	NH2	1	None,None	NH2,1
paranh22	para	NH2	2	NH2	2	NH2,2	NH2,2
paranh23	para	NH2	3	NH2	2	NH2,3	NH2,2
paranh24	para	NH2	4	NH2	2	NH2,4	NH2,2
paranh2	para	None	None	NH2	2	None,None	NH2,2
paranh33	para	NH2	3	NH2	3	NH2,3	NH2,3
paranh34	para	NH2	4	NH2	3	NH2,4	NH2,3
paranh3	para	None	None	NH2	3	None,None	NH2,3
paranh44	para	NH2	4	NH2	4	NH2,4	NH2,4
paranh4	para	None	None	NH2	4	None,None	NH2,4
paranhbr12	para	NH2	1	Br	2	NH2,1	Br,2
paranhbr13	para	NH2	1	Br	3	NH2,1	Br,3
paranhbr14	para	NH2	1	Br	4	NH2,1	Br,4
paranhbr23	para	NH2	2	Br	3	NH2,2	Br,3
paranhbr24	para	NH2	2	Br	4	NH2,2	Br,4
paranhbr34	para	NH2	3	Br	4	NH2,3	Br,4
paranhno11	para	NH2	1	NO2	1	NH2,1	NO2,1
paranhno12	para	NH2	1	NO2	2	NH2,1	NO2,2
paranhno13	para	NH2	1	NO2	3	NH2,1	NO2,3
paranhno14	para	NH2	1	NO2	4	NH2,1	NO2,4
paranhno22	para	NH2	2	NO2	2	NH2,2	NO2,2
paranhno23	para	NH2	2	NO2	3	NH2,2	NO2,3
paranhno24	para	NH2	2	NO2	4	NH2,2	NO2,4
paranhno33	para	NH2	3	NO2	3	NH2,3	NO2,3
paranhno34	para	NH2	3	NO2	4	NH2,3	NO2,4
paranhno44	para	NH2	4	NO2	4	NH2,4	NO2,4
parano11	para	NO2	1	NO2	1	NO2,1	NO2,1
parano12	para	NO2	2	NO2	1	NO2,2	NO2,1
parano14	para	NO2	4	NO2	1	NO2,4	NO2,1
parano1	para	None	None	NO2	1	None,None	NO2,1

parano22	para	NO2	2	NO2	2	NO2,2	NO2,2
parano23	para	NO2	3	NO2	2	NO2,3	NO2,2
parano24	para	NO2	4	NO2	2	NO2,4	NO2,2
parano2	para	None	None	NO2	2	None,None	NO2,2
parano33	para	NO2	3	NO2	3	NO2,3	NO2,3
parano34	para	NO2	4	NO2	3	NO2,4	NO2,3
parano3	para	None	None	NO2	3	None,None	NO2,3
parano44	para	NO2	4	NO2	4	NO2,4	NO2,4
parano4	para	None	None	NO2	4	None,None	NO2,4
paranobr12	para	NO2	1	Br	2	NO2,1	Br,2
paranobr13	para	NO2	1	Br	3	NO2,1	Br,3
paranobr14	para	NO2	1	Br	4	NO2,1	Br,4
paranobr23	para	NO2	2	Br	3	NO2,2	Br,3
paranobr24	para	NO2	2	Br	4	NO2,2	Br,4
paranobr34	para	NO2	3	Br	4	NO2,3	Br,4
paranonh12	para	NO2	1	NH2	2	NO2,1	NH2,2
paranonh13	para	NO2	1	NH2	3	NO2,1	NH2,3
paranonh14	para	NO2	1	NH2	4	NO2,1	NH2,4
paranonh23	para	NO2	2	NH2	3	NO2,2	NH2,3
paranonh24	para	NO2	2	NH2	4	NO2,2	NH2,4
paranonh34	para	NO2	3	NH2	4	NO2,3	NH2,4
para	para	None	None	None	None	None,None	None,None
