# Supplementary material for: Comprehensive Empirical Model of Substitution—Influence on Hydrogen Bonding in Aromatic Schiff Bases
Source: Int J Mol Sci. 2022 Oct 18;23(20):12439. doi: 10.3390/ijms232012439 (PMC9604547; doi:10.3390/ijms232012439)

Supplement SB: Selected parameters

Comprehensive empirical model of substitution – influence on hydrogen bonding in aromatic Schiff bases

Katarzyna M. Krupka[1], *Michał Pocheć*[1], *Jarosław J. Panek*[1] *and Aneta Jezierska*[1]*

[1]Faculty of Chemistry, University of Wrocław, ul. F. Joliot-Curie 14, 50-383 Wrocław, Poland

Correspondence should be addressed to: aneta.jezierska@chem.uni.wroc.pl.

Table SB1. Statistical description of activation energies for ortho group – proximal effect in the gas phase.

Sub.	Mean activation energy [kcal/mol]	Standard deviation	Min. Value	Max. Value
None	7.228	0.987	5.910	9.250
NO2,4	7.114	0.820	5.977	8.645
NO2,3	7.385	0.949	5.998	9.303
NO2,2	7.425	0.968	5.894	9.238
NO2,1	7.797	0.928	6.429	9.688
NH2,4	7.225	0.981	5.788	9.100
NH2,3	7.107	0.923	5.650	9.069
NH2,2	7.428	0.949	5.975	9.274
NH2,1	7.242	0.869	5.856	9.057
Br,4	7.221	0.823	5.979	8.762
Br,3	7.451	0.901	6.162	9.240
Br,2	7.374	0.928	5.929	9.272
Br,1	7.457	0.942	5.981	9.411

Table SB2. Statistical description of activation energies for ortho group – distal effect in the gas phase.

Sub.	Mean activation energy [kcal/mol]	Standard deviation	Min. Value	Max. Value
Br,1	6.449	0.167	6.204	6.684
Br,2	8.105	0.259	7.785	8.619
Br,3	7.700	0.284	7.347	8.200
Br,4	6.858	0.254	6.625	7.635
NH2,1	7.410	0.246	6.934	7.675
NH2,2	9.178	0.266	8.645	9.688
NH2,3	7.229	0.302	6.838	7.823
NH2,4	7.359	0.201	6.981	7.793
NO2,1	6.140	0.159	5.856	6.429
NO2,2	6.852	0.214	6.573	7.272
NO2,3	7.946	0.253	7.524	8.500
NO2,4	5.992	0.252	5.650	6.702
None	8.219	0.264	7.795	8.525

Table SB3. Statistical description of activation energies for meta group – proximal effect in the gas phase.

Sub.	Mean activation energy [kcal/mol]	Standard deviation	Min. Value	Max. Value
None	7.359	0.890	5.938	9.119
NO2,4	7.683	0.877	6.216	9.361
NO2,3	7.620	0.876	6.199	9.303
NO2,2	7.575	0.879	6.150	9.274
NO2,1	7.474	0.885	6.045	9.191
NH2,4	7.314	0.892	5.900	9.066
NH2,3	7.181	0.894	5.779	8.959
NH2,2	7.312	0.890	5.904	9.053
NH2,1	7.316	0.905	5.882	9.093
Br,4	7.501	0.885	6.058	9.215
Br,3	7.447	0.885	6.018	9.169
Br,2	7.452	0.885	6.029	9.182
Br,1	7.417	0.890	5.981	9.155

Table SB4. Statistical description of activation energies for meta group – distal effect in the gas phase.

Sub.	Mean activation energy [kcal/mol]	Standard deviation	Min. Value	Max. Value
Br,1	6.534	0.140	6.290	6.766
Br,2	8.175	0.145	7.917	8.417
Br,3	7.746	0.143	7.478	7.986
Br,4	7.273	0.150	7.015	7.534
NH2,1	7.311	0.159	7.039	7.627
NH2,2	9.165	0.110	8.959	9.361
NH2,3	7.276	0.113	7.060	7.480
NH2,4	7.408	0.144	7.159	7.646
NO2,1	6.007	0.129	5.779	6.216
NO2,2	6.868	0.174	6.544	7.155
NO2,3	8.108	0.149	7.837	8.371
NO2,4	6.364	0.161	6.106	6.672
None	8.414	0.134	8.171	8.653

Table SB5. Statistical description of activation energies for para group – proximal effect in the gas phase.

Sub.	Mean activation energy [kcal/mol]	Standard deviation	Min. Value	Max. Value
None	7.181	0.903	5.688	8.946
NO2,4	7.441	0.899	5.915	9.155
NO2,3	7.592	0.798	6.379	9.174
NO2,2	7.495	0.891	5.957	9.190
NO2,1	7.372	0.902	5.973	9.143
NH2,4	7.154	0.904	5.666	8.924
NH2,3	7.010	0.907	5.522	8.800
NH2,2	7.125	0.904	5.639	8.892
NH2,1	7.128	0.899	5.621	8.871
Br,4	7.291	0.900	5.775	9.019
Br,3	7.286	0.896	5.783	9.011
Br,2	7.311	0.900	5.802	9.056
Br,1	7.264	0.903	5.766	9.018

Table SB6. Statistical description of activation energies for para group – distal effect in the gas phase.

Sub.	Mean activation energy [kcal/mol]	Standard deviation	Min. Value	Max. Value
Br,1	6.397	0.144	6.148	6.608
Br,2	8.008	0.151	7.737	8.233
Br,3	7.583	0.151	7.329	7.809
Br,4	7.106	0.159	6.827	7.335
NH2,1	7.226	0.185	6.924	7.480
NH2,2	9.015	0.125	8.800	9.190
NH2,3	7.140	0.122	6.916	7.311
NH2,4	7.285	0.153	7.016	7.509
NO2,1	5.759	0.140	5.522	5.973
NO2,2	6.678	0.184	6.354	6.950
NO2,3	7.917	0.157	7.641	8.147
NO2,4	6.180	0.159	5.912	6.437
None	8.257	0.146	8.002	8.473

Table SB7. Statistical description of activation energies for ortho group – proximal effect in PCM.

Sub.	Mean activation energy [kcal/mol]	Standard deviation	Min. value	Max. Value
None	7.228	0.987	5.910	9.250
NO2,4	7.147	0.794	5.977	8.645
NO2,3	7.385	0.949	5.998	9.303
NO2,2	7.425	0.968	5.894	9.238
NO2,1	7.797	0.928	6.429	9.688
NH2,4	7.228	0.939	5.788	9.100
NH2,3	7.107	0.923	5.650	9.069
NH2,2	7.428	0.949	5.975	9.274
NH2,1	7.242	0.869	5.856	9.057
Br,4	7.221	0.823	5.979	8.762
Br,3	7.318	0.912	5.850	9.240
Br,2	7.374	0.928	5.929	9.272
Br,1	7.457	0.942	5.981	9.411

Table SB8. Statistical description of activation energies for ortho group – distal effect in PCM.

Sub.	Mean activation energy [kcal/mol]	Standard deviation	Min. value	Max. Value
Br,1	6.449	0.167	6.204	6.684
Br,2	8.105	0.259	7.785	8.619
Br,3	7.663	0.278	7.268	8.200
Br,4	6.858	0.254	6.625	7.635
NH2,1	7.410	0.246	6.934	7.675
NH2,2	9.178	0.266	8.645	9.688
NH2,3	7.229	0.302	6.838	7.823
NH2,4	7.329	0.219	6.976	7.793
NO2,1	6.140	0.159	5.856	6.429
NO2,2	6.852	0.214	6.573	7.272
NO2,3	7.946	0.253	7.524	8.500
NO2,4	5.981	0.245	5.650	6.702
None	8.219	0.264	7.795	8.525

Table SB9. Statistical description of activation energies for meta group – proximal effect in PCM.

Sub.	Mean activation energy [kcal/mol]	Standard deviation	Min. value	Max. Value
None	7.161	0.869	5.848	8.871
NO2,4	7.455	0.855	6.102	9.071
NO2,3	7.404	0.858	6.092	9.037
NO2,2	7.367	0.859	6.055	9.008
NO2,1	7.279	0.864	5.963	8.951
NH2,4	7.124	0.866	5.815	8.825
NH2,3	6.995	0.864	5.694	8.705
NH2,2	7.118	0.870	5.816	8.811
NH2,1	7.120	0.883	5.788	8.848
Br,4	7.292	0.864	5.963	8.955
Br,3	7.243	0.863	5.921	8.905
Br,2	7.248	0.866	5.939	8.930
Br,1	7.222	0.869	5.896	8.901

Table SB10. Statistical description of activation energies for meta group – distal effect in the gas phase.

Sub.	Mean activation energy [kcal/mol]	Standard deviation	Min. value	Max. Value
Br,1	6.393	0.129	6.174	6.595
Br,2	7.969	0.135	7.727	8.186
Br,3	7.541	0.134	7.284	7.763
Br,4	7.052	0.143	6.797	7.281
NH2,1	7.098	0.155	6.839	7.401
NH2,2	8.909	0.100	8.705	9.071
NH2,3	7.057	0.107	6.844	7.236
NH2,4	7.205	0.123	6.984	7.413
NO2,1	5.915	0.123	5.694	6.102
NO2,2	6.664	0.157	6.385	6.920
NO2,3	7.910	0.142	7.640	8.149
NO2,4	6.105	0.137	5.888	6.377
None	8.209	0.128	7.967	8.426

Table SB11. Statistical description of activation energies for para group – proximal effect in PCM.

Sub.	Mean activation energy [kcal/mol]	Standard deviation	Min. value	Max. Value
None	7.012	0.880	5.633	8.728
NO2,4	7.254	0.877	5.851	8.913
NO2,3	7.280	0.869	5.893	8.925
NO2,2	7.298	0.867	5.897	8.948
NO2,1	7.226	0.870	5.837	8.896
NH2,4	6.990	0.879	5.612	8.708
NH2,3	6.852	0.884	5.484	8.609
NH2,2	6.964	0.883	5.584	8.683
NH2,1	6.968	0.882	5.559	8.685
Br,4	7.120	0.873	5.726	8.797
Br,3	7.111	0.874	5.719	8.787
Br,2	7.134	0.876	5.740	8.822
Br,1	7.092	0.879	5.704	8.798

Table SB12. Statistical description of activation energies for para group – distal effect in PCM.

Sub.	Mean activation energy [kcal/mol]	Standard deviation	Min. value	Max. Value
Br,1	6.277	0.134	6.044	6.471
Br,2	7.828	0.140	7.574	8.027
Br,3	7.402	0.138	7.159	7.597
Br,4	6.903	0.151	6.630	7.121
NH2,1	7.034	0.175	6.739	7.274
NH2,2	8.792	0.106	8.609	8.948
NH2,3	6.943	0.109	6.733	7.084
NH2,4	7.109	0.135	6.870	7.302
NO2,1	5.711	0.133	5.484	5.897
NO2,2	6.509	0.165	6.215	6.755
NO2,3	7.757	0.147	7.479	7.950
NO2,4	5.956	0.139	5.707	6.170
None	8.080	0.137	7.835	8.275

Figure SB1. Differences of charge distribution from the non-substituted compound for the ortho group in the gas phase – Br substitution on proximal ring.

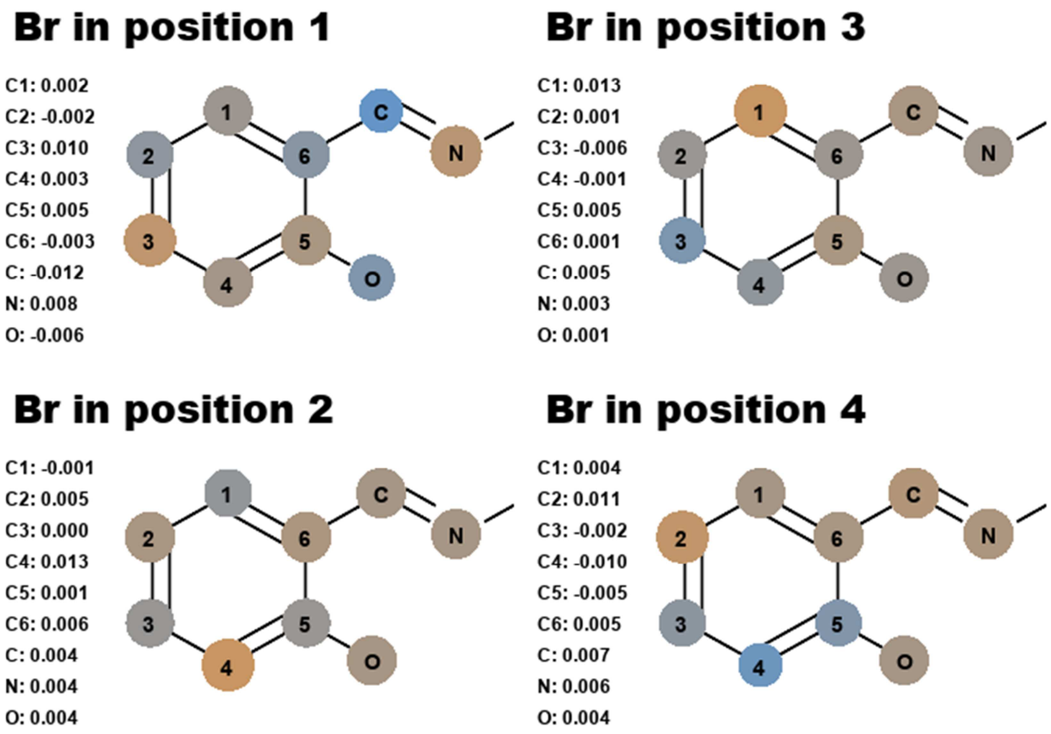

Figure SB2. Differences of charge distribution from the non-substituted compound for the ortho group in the gas phase – NO_2 substitution on proximal ring.

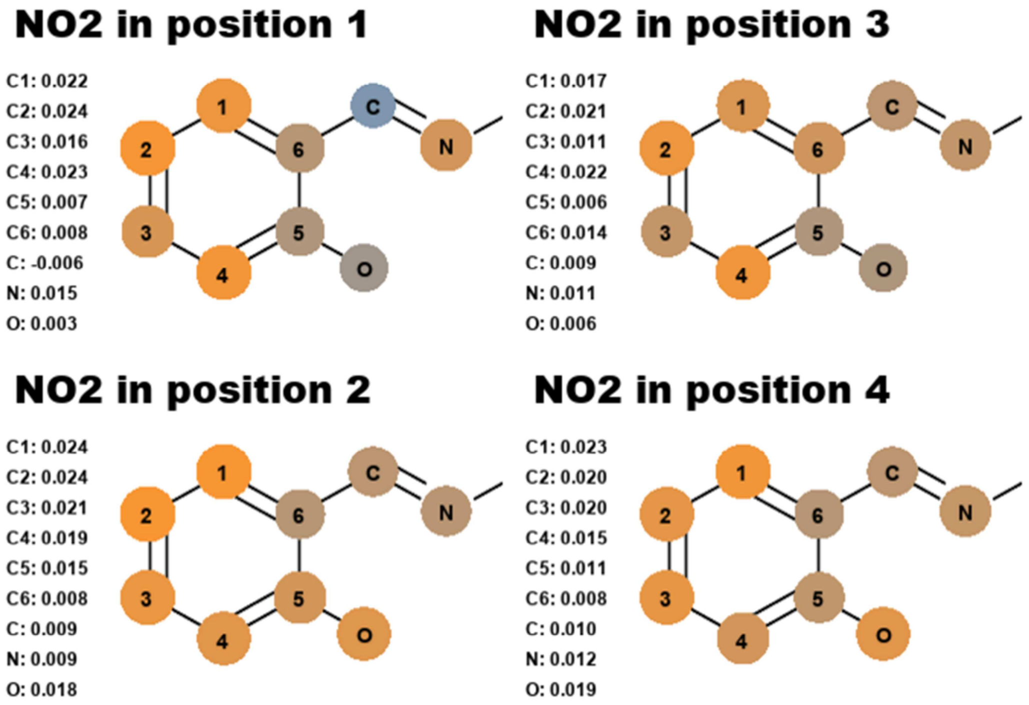

Figure SB3. Differences of charge distribution from the non-substituted compound for the ortho group in the gas phase – NH_2 substitution on proximal ring.

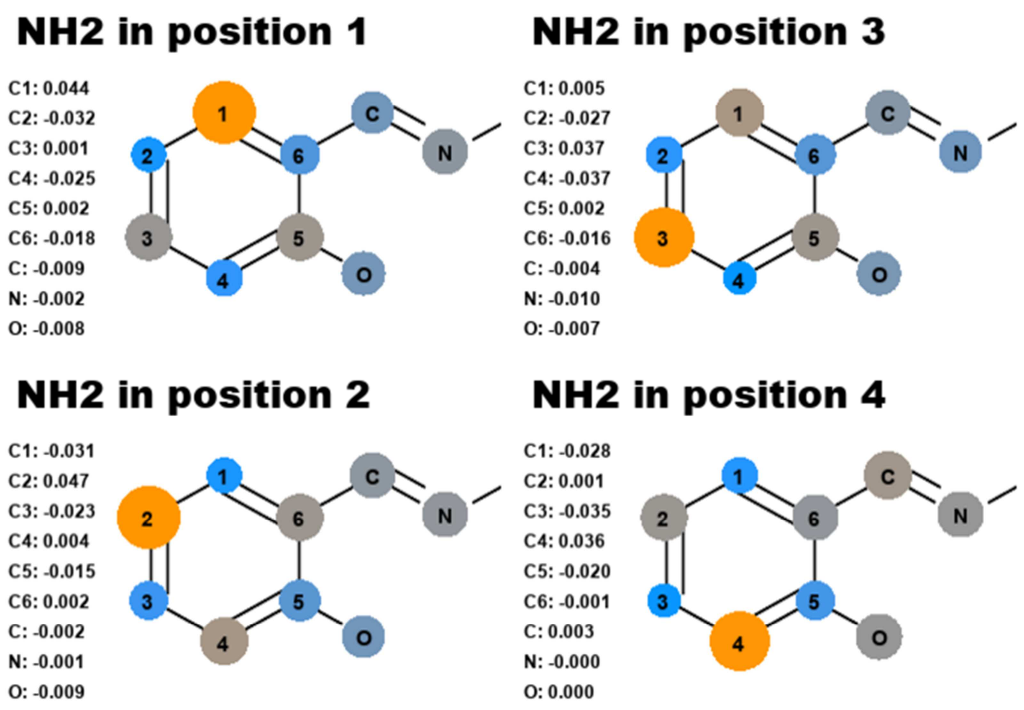

Figure SB4. Differences of charge distribution from the non-substituted compound for the meta group in the gas phase – Br substitution on proximal ring.

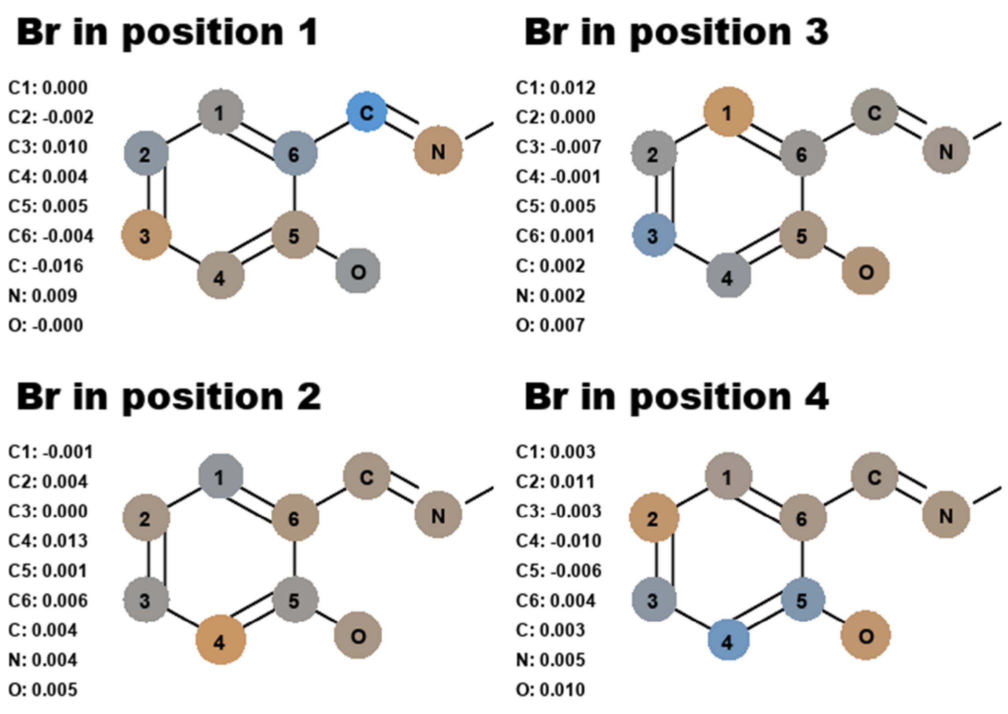

Figure SB5. Differences of charge distribution from the non-substituted compound for the meta group in the gas phase – NO_2 substitution on proximal ring.

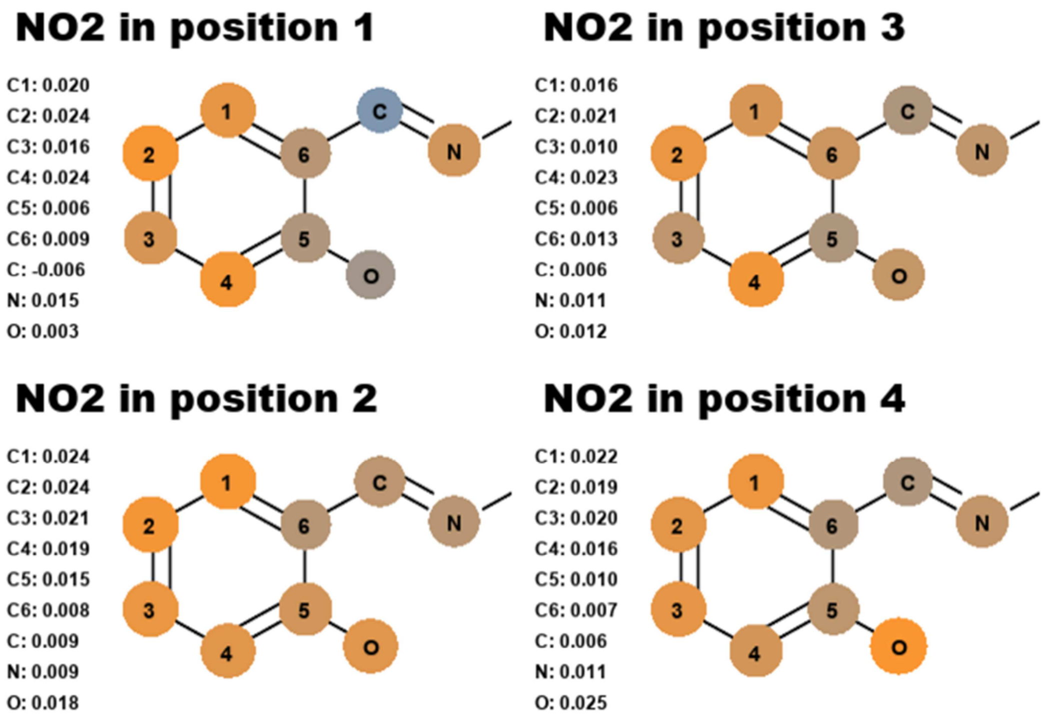

Figure SB6. Differences of charge distribution from the non-substituted compound for the meta group in the gas phase – NH_2 substitution on proximal ring.

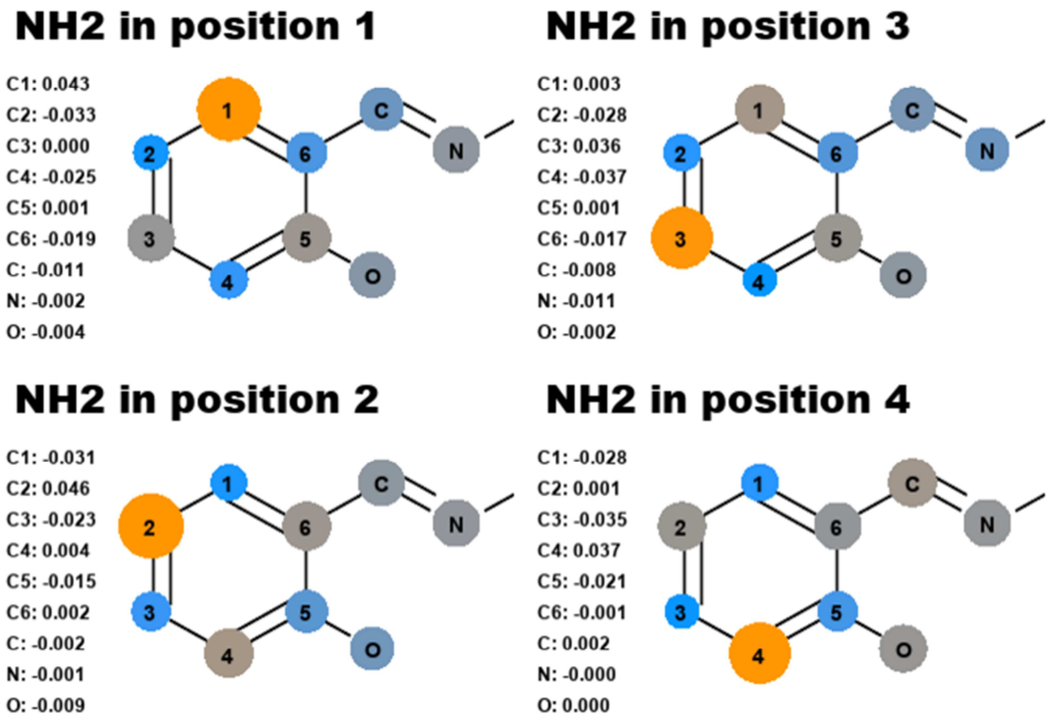

Figure SB7. Differences of charge distribution from the non-substituted compound for the para group in the gas phase – Br substitution.

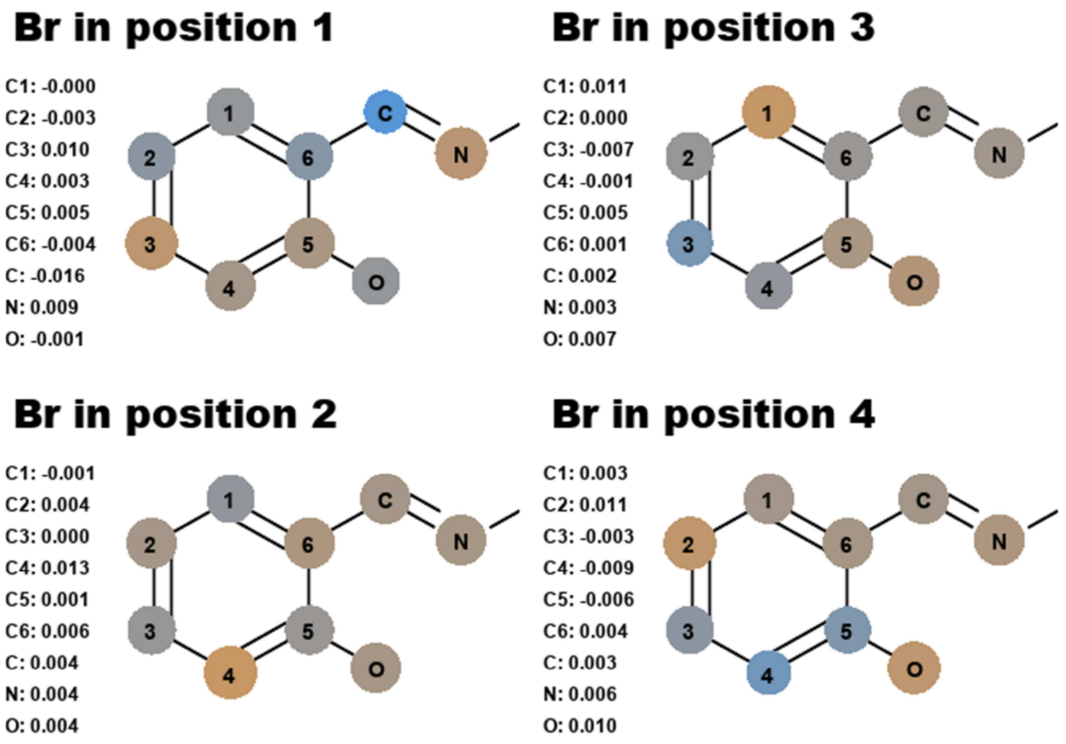

Figure SB8. Differences of charge distribution from the non-substituted compound for the para group in the gas phase – NO_2 substitution.

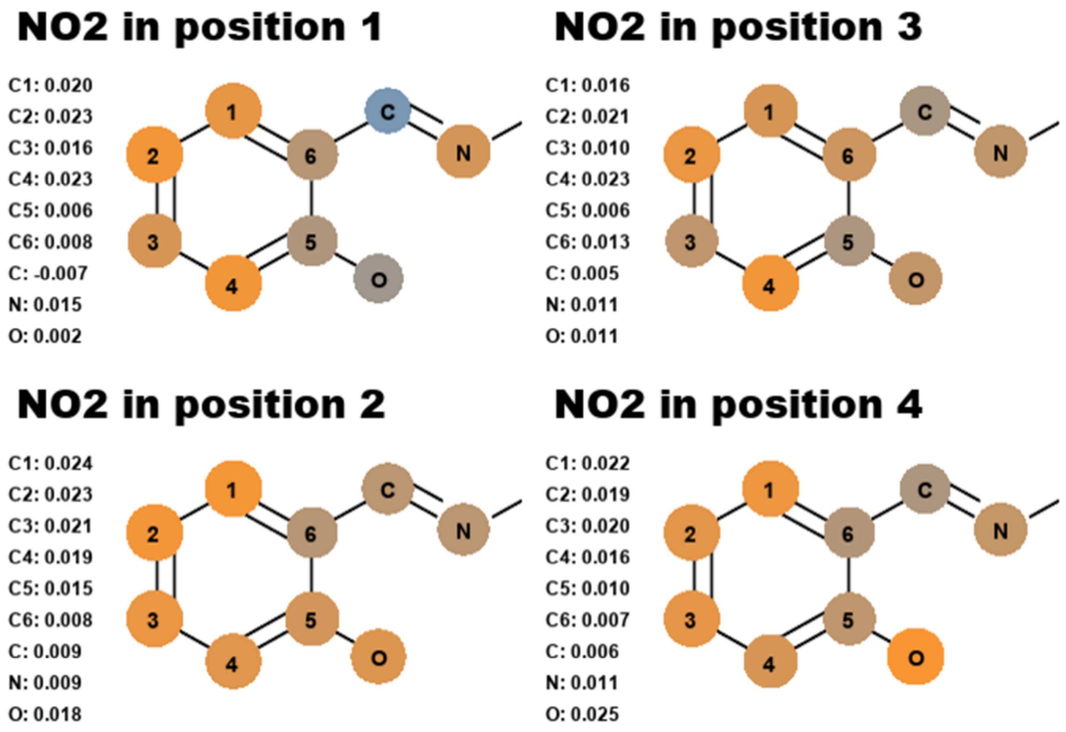

Figure SB9. Differences of charge distribution from the non-substituted compound for the para group in the gas phase – NH_2 substitution.

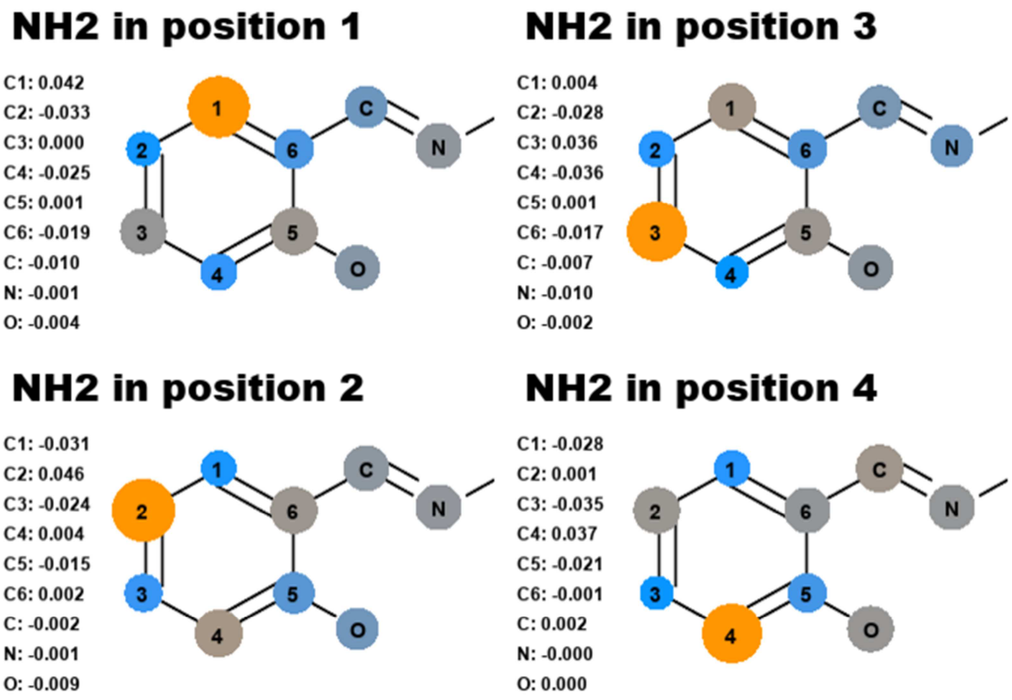

Figure SB10. Additional parameters distribution in different substitution patterns for ortho compounds group in the gas phase.

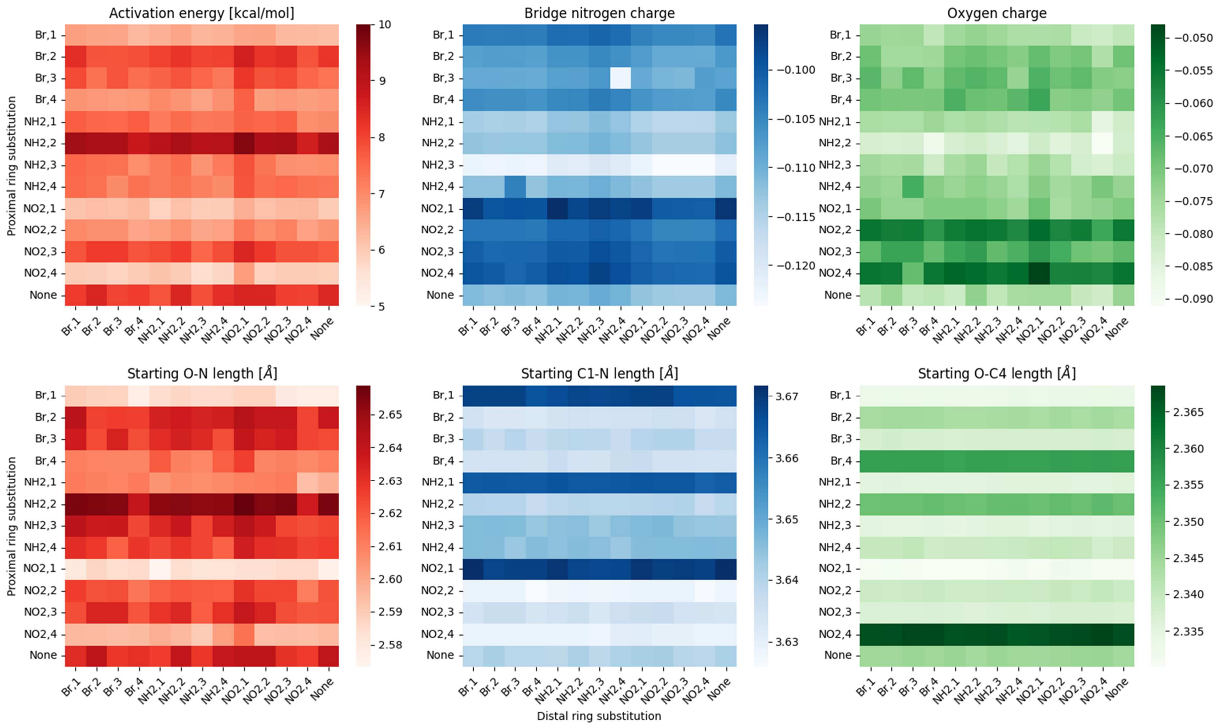

Figure SB11. Additional parameters distribution in different substitution patterns for meta compounds group in the gas phase.

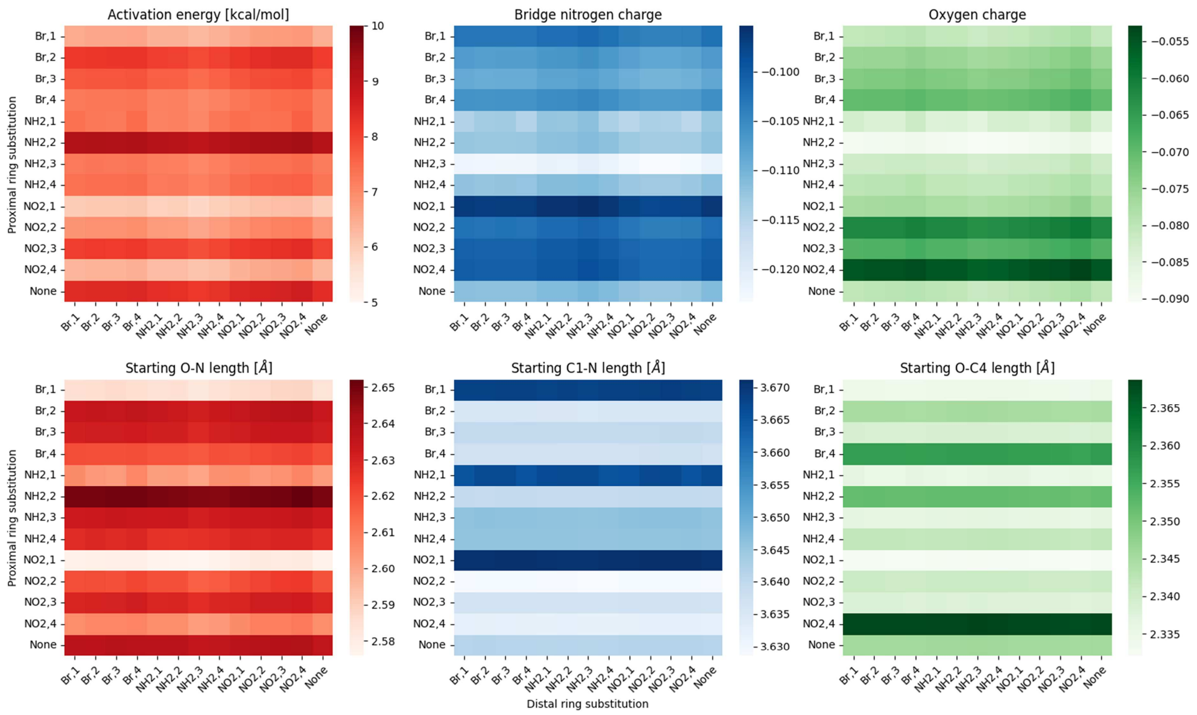

Figure SB12. Additional parameters distribution in different substitution patterns for para compounds group in the gas phase.

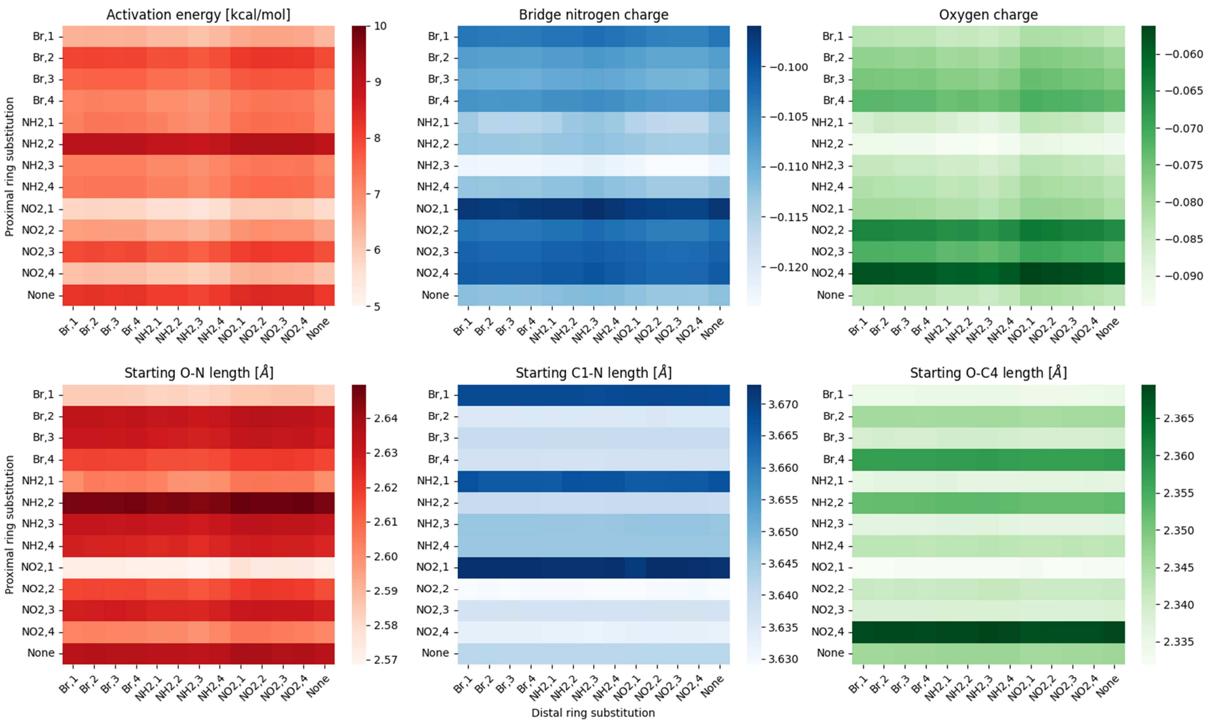

Figure SB13. Heatmaps of the activation energy (left), second minimum (middle) and the difference between them (right) in different substitution patterns for para compounds group in PCM.

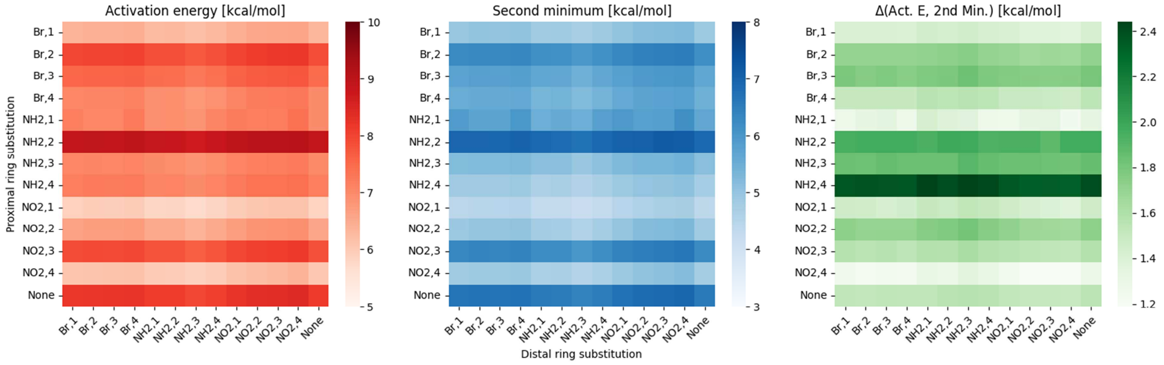

Figure SB14. Heatmaps of the activation energy (left), second minimum (middle) and the difference between them (right) in different substitution patterns for meta compounds group in PCM.

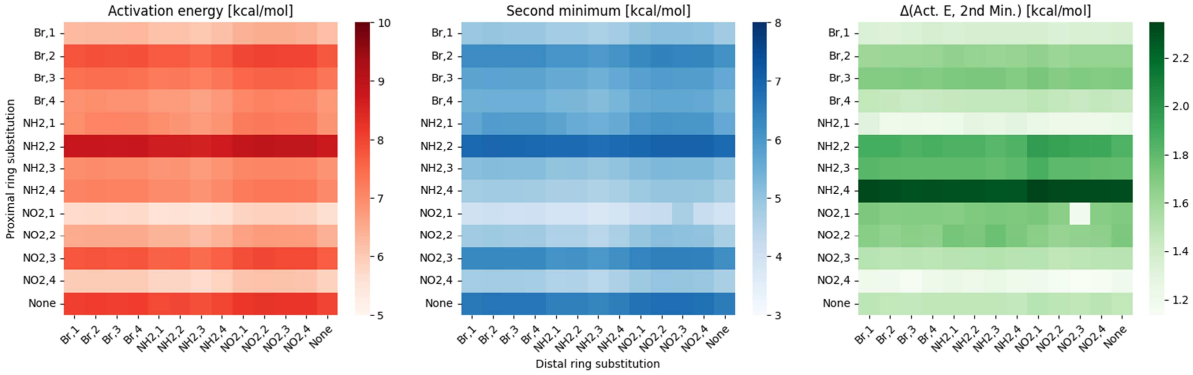

Figure SB15. Heatmaps of the activation energy (left), second minimum (middle) and the difference between them (right) in different substitution patterns for ortho compounds group in PCM.

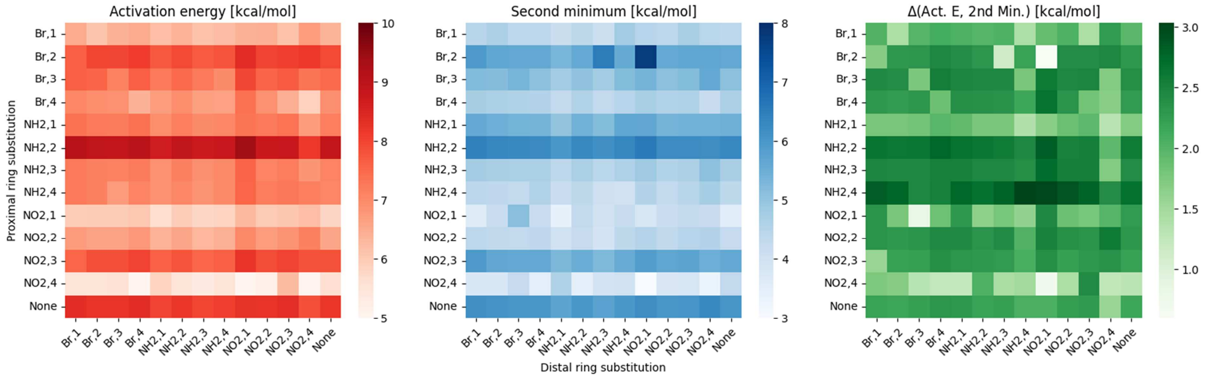

Figure SB16. Distribution of residuals for the model assessment – green for activation energy and yellow for the second minimum.

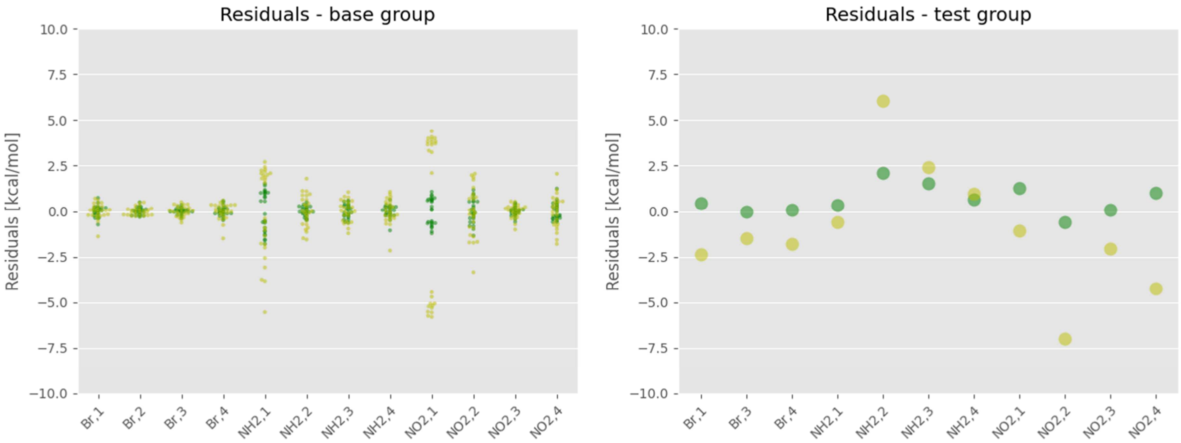

Supplement: Supplementary file 1 [file ijms-23-12439-s001.zip › Supplement B.pdf]
