# Supplementary material for: Comprehensive Empirical Model of Substitution—Influence on Hydrogen Bonding in Aromatic Schiff Bases
Source: Int J Mol Sci. 2022 Oct 18;23(20):12439. doi: 10.3390/ijms232012439 (PMC9604547; doi:10.3390/ijms232012439)

Supplement SC: Topological analysis

Comprehensive empirical model of substitution – influence on hydrogen bonding in aromatic Schiff bases

*Katarzyna M. Krupka[1], Michał Pocheć[1], Jarosław J. Panek[1] and Aneta Jezierska[1]**

[1]Faculty of Chemistry, University of Wrocław, ul. F. Joliot-Curie 14, 50-383 Wrocław, Poland

Correspondence should be addressed to: aneta.jezierska@chem.uni.wroc.pl.

Table SC1. QTAIM Bond Critical Point (BCP) properties for selected bonds in the nonsubstituted para isomer.

	Gas Phase		PCM	
**BCP**	**ρBCP [e*a^{-3}]**	**∇^2ρBCP [e*a^{-5}]**	**ρBCP [e*a^{-3}]**	**∇^2ρBCP [e*a^{-5}]**
**Molecular form**				
O-H	0.345	-2.621	0.340	-2.566
H…N	0.046	0.108	0.050	0.110
**Proton-transferred form**				
O…H	0.026	0.082	0.026	0.081
H-N	0.353	-2.152	0.354	-2.160

Table SC2. QTAIM Bond Critical Point (BCP) properties for selected bonds in the para isomer monosubstituted with the NH_2 group in the proximal position 1.

	Gas Phase		PCM	
**BCP**	**ρBCP [e*a^{-3}]**	**∇^2ρBCP [e*a^{-5}]**	**ρBCP [e*a^{-3}]**	**∇^2ρBCP [e*a^{-5}]**
**Molecular form**				
O-H	0.342	-2.586	0.337	-2.519
H…N	0.051	0.112	0.056	0.113
**Proton-transferred form**				
O…H	0.026	0.082	0.025	0.081
H-N	0.356	-2.172	0.357	-2.182

Table SC3. QTAIM Bond Critical Point (BCP) properties for selected bonds in the para isomer monosubstituted with the NH_2 group in the proximal position 4.

	**Gas Phase**		**PCM**	
**BCP**	**ρBCP [$e*a^{-3}$]**	**∇^2ρBCP [$e*a^{-5}$]**	**ρBCP [$e*a^{-3}$]**	**∇^2ρBCP [$e*a^{-5}$]**
**Molecular form**				
O-H	0.343	-2.605	0.340	-2.562
H…N	0.047	0.108	0.050	0.109
**Proton-transferred form**				
O…H	0.026	0.083	0.025	0.082
H-N	0.354	-2.154	0.355	-2.162

Table SC4. QTAIM Bond Critical Point (BCP) properties for selected bonds in the para isomer monosubstituted with the NO_2 group in the proximal position 1.

	**Gas Phase**		**PCM**	
**BCP**	**ρBCP [$e*a^{-3}$]**	**∇^2ρBCP [$e*a^{-5}$]**	**ρBCP [$e*a^{-3}$]**	**∇^2ρBCP [$e*a^{-5}$]**
**Molecular form**				
O-H	0.336	-2.525	0.333	-2.492
H…N	0.056	0.112	0.058	0.112
**Proton-transferred form**				
O…H	0.025	0.082	0.025	0.081
H-N	0.357	-2.206	0.358	-2.210

Table SC5. QTAIM Bond Critical Point (BCP) properties for selected bonds in the para isomer monosubstituted with the NO_2 group in the proximal position 4.

	**Gas Phase**		**PCM**	
**BCP**	**ρBCP [$e*a^{-3}$]**	**∇^2ρBCP [$e*a^{-5}$]**	**ρBCP [$e*a^{-3}$]**	**∇^2ρBCP [$e*a^{-5}$]**
**Molecular form**				
O-H	0.338	-2.549	0.329	-2.444
H…N	0.052	0.109	0.058	0.109
**Proton-transferred form**				
O…H	0.025	0.081	0.025	0.079
H-N	0.355	-2.181	0.357	-2.195

Table SC6. QTAIM Bond Critical Point (BCP) properties for selected bonds in the para isomer monosubstituted with the Br atom in the proximal position 1.

	Gas Phase		PCM	
BCP	ρBCP [e*a^{-3}]	∇^2ρBCP [e*a^{-5}]	ρBCP [e*a^{-3}]	∇^2ρBCP [e*a^{-5}]
Molecular form				
O-H	0.338	-2.547	0.334	-2.499
H…N	0.054	0.112	0.057	0.112
Proton-transferred form				
O…H	0.025	0.082	0.025	0.081
H-N	0.358	-2.203	0.358	-2.210

Table SC7. QTAIM Bond Critical Point (BCP) properties for selected bonds in the para isomer monosubstituted with the Br atom in the proximal position 4.

	Gas Phase		PCM	
BCP	ρBCP [e*a^{-3}]	∇^2ρBCP [e*a^{-5}]	ρBCP [e*a^{-3}]	∇^2ρBCP [e*a^{-5}]
Molecular form				
O-H	0.341	-2.586	0.335	-2.508
H…N	0.049	0.109	0.054	0.110
Proton-transferred form				
O…H	0.026	0.082	0.025	0.081
H-N	0.355	-2.173	0.356	-2.184

Table SC8. QTAIM net atomic charges for atoms forming quasi-ring in the nonsubstituted para isomer.

Net atomic charge [e]	Gas Phase	PCM
Molecular form		
O	-1.200	-1.198
H	0.667	0.666
N	-1.133	-1.128
C_B	0.626	0.628
C6	-0.028	-0.028
C5	0.601	0.580
SUM	-0.467	-0.480
Proton-transferred form		
O	-1.156	-1.151
H	0.512	0.509
N	-1.208	-1.203
C_B	0.479	0.485
C6	-0.044	-0.047
C5	0.900	0.894
SUM	-0.517	-0.513

Table SC9. QTAIM net atomic charges for atoms forming quasi-ring in the para isomer monosubstituted with the NH_2 group in the proximal position 1.

**Net atomic charge [e]**	**Gas Phase**	**PCM**
**Molecular form**		
O	-1.205	-1.188
H	0.664	0.660
N	-1.138	-1.115
C_B	0.626	0.617
C6	-0.052	-0.043
C5	0.617	0.580
SUM	-0.488	-0.489
**Proton-transferred form**		
O	-1.151	-1.149
H	0.505	0.502
N	-1.200	-1.188
C_B	0.507	0.480
C6	-0.068	-0.070
C5	0.889	0.890
SUM	-0.518	-0.535

Table SC10. QTAIM net atomic charges for atoms forming quasi-ring in the para isomer monosubstituted with the NH_2 group in the proximal position 4.

**Net atomic charge [e]**	**Gas Phase**	**PCM**
**Molecular form**		
O	-1.196	-1.185
H	0.648	0.653
N	-1.139	-1.132
C_B	0.650	0.657
C6	-0.021	-0.030
C5	0.578	0.591
SUM	-0.480	-0.446
**Proton-transferred form**		
O	-1.168	-1.155
H	0.479	0.512
N	-1.181	-1.231
C_B	0.508	0.511
C6	-0.037	-0.051
C5	0.869	0.845
SUM	-0.530	-0.569

Table SC11. QTAIM net atomic charges for atoms forming quasi-ring in the para isomer monosubstituted with the NO_2 group in the proximal position 1.

**Net atomic charge [e]**	**Gas Phase**	**PCM**
**Molecular form**		
O	-1.202	-1.187
H	0.664	0.650
N	-1.111	-1.123
C_B	0.613	0.651
C6	-0.019	-0.011
C5	0.651	0.634
SUM	-0.404	-0.386
**Proton-transferred form**		
O	-1.146	-1.143
H	0.503	0.505
N	-1.184	-1.184
C_B	0.529	0.529
C6	-0.038	-0.041
C5	0.899	0.898
SUM	-0.437	-0.436

Table SC12. QTAIM net atomic charges for atoms forming quasi-ring in the para isomer monosubstituted with the NO_2 group in the proximal position 4.

**Net atomic charge [e]**	**Gas Phase**	**PCM**
**Molecular form**		
O	-1.173	-1.180
H	0.654	0.676
N	-1.136	-1.163
C_B	0.655	0.646
C6	-0.020	-0.027
C5	0.700	0.656
SUM	-0.320	-0.392
**Proton-transferred form**		
O	-1.127	-1.116
H	0.508	0.509
N	-1.245	-1.220
C_B	0.526	0.500
C6	-0.042	-0.039
C5	0.955	0.938
SUM	-0.425	-0.428

Table SC13. QTAIM net atomic charges for atoms forming quasi-ring in the para isomer monosubstituted with the Br atom in the proximal position 1.

Net atomic charge [e]	Gas Phase	PCM
**Molecular form**		
O	-1.189	-1.196
H	0.649	0.663
N	-1.127	-1.146
C_B	0.636	0.648
C6	-0.005	-0.028
C5	0.629	0.620
SUM	-0.407	-0.439
**Proton-transferred form**		
O	-1.148	-1.148
H	0.497	0.502
N	-1.200	-1.201
C_B	0.524	0.524
C6	-0.042	-0.043
C5	0.891	0.891
SUM	-0.478	-0.475

Table SC14. QTAIM net atomic charges for atoms forming quasi-ring in the para isomer monosubstituted with the Br atom in the proximal position 4.

Net atomic charge [e]	Gas Phase	PCM
**Molecular form**		
O	-1.186	-1.178
H	0.651	0.647
N	-1.140	-1.137
C_B	0.653	0.645
C6	-0.022	-0.014
C5	0.634	0.679
SUM	-0.410	-0.358
**Proton-transferred form**		
O	-1.132	-1.116
H	0.512	0.509
N	-1.178	-1.220
C_B	0.484	0.450
C6	-0.019	-0.039
C5	0.913	0.938
SUM	-0.420	-0.478

Figure SC1. QTAIM topology maps for the nonsubstituted para isomer: a) molecular form in the gas phase, b) proton-transferred form in the gas phase, c) molecular form in PCM, d) proton-transferred form in PCM. The orange and yellow dots mark the BCPs and RCPs, respectively. Yellow lines pinpoint the intramolecular interaction paths.

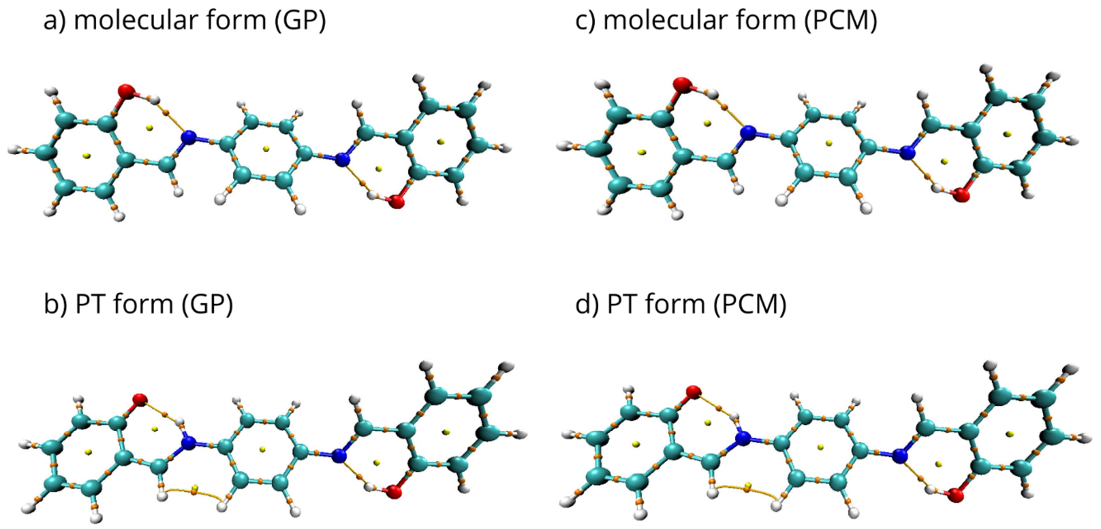

Figure SC2. QTAIM topology maps for the para isomer monosubstituted with the NH_2 group in the proximal position 1: a) molecular form in the gas phase, b) proton-transferred form in the gas phase, c) molecular form in PCM, d) proton-transferred form in PCM. The orange and yellow dots mark the BCPs and RCPs, respectively. Yellow lines pinpoint the intramolecular interaction paths.

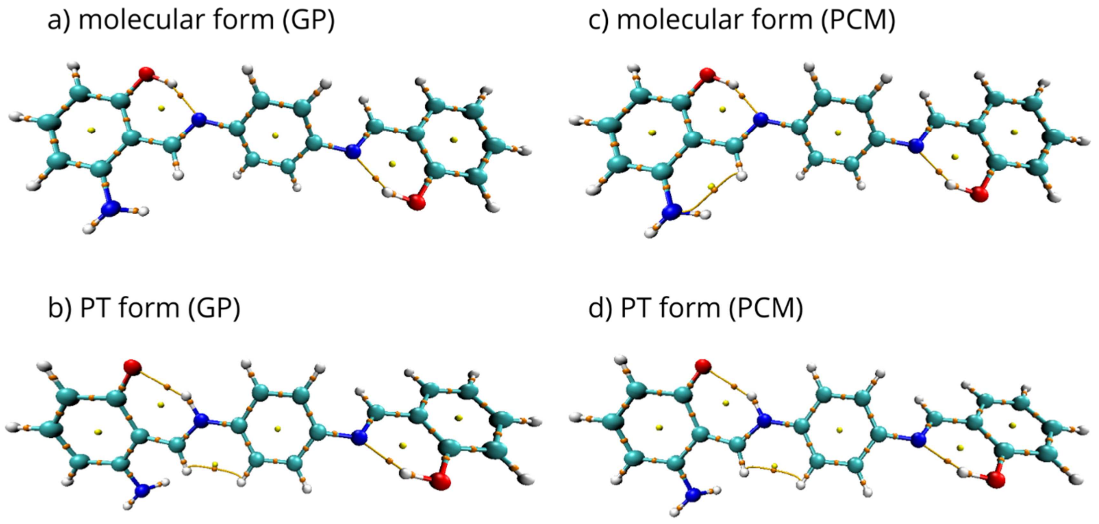

Figure SC3. QTAIM topology maps for the para isomer monosubstituted with the NH_2 group in the proximal position 4: a) molecular form in the gas phase, b) proton-transferred form in the gas phase, c) molecular form in PCM, d) proton-transferred form in PCM. The orange and yellow dots mark the BCPs and RCPs, respectively. Yellow lines pinpoint the intramolecular interaction paths.

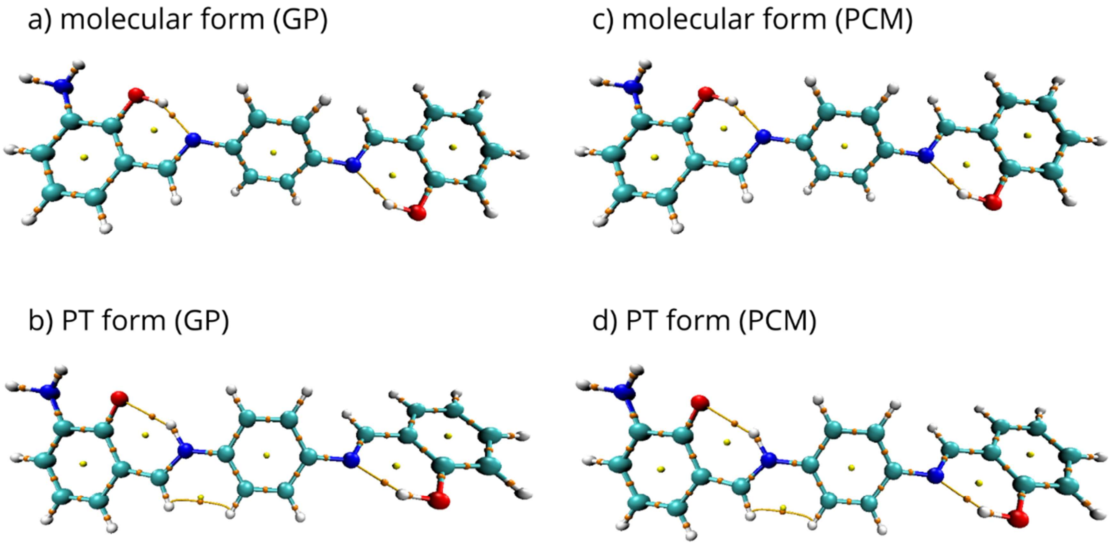

Figure SC4. QTAIM topology maps for the para isomer monosubstituted with the NO_2 group in the proximal position 1: a) molecular form in the gas phase, b) proton-transferred form in the gas phase, c) molecular form in PCM, d) proton-transferred form in PCM. The orange and yellow dots mark the BCPs and RCPs, respectively. Yellow lines pinpoint the intramolecular interaction paths.

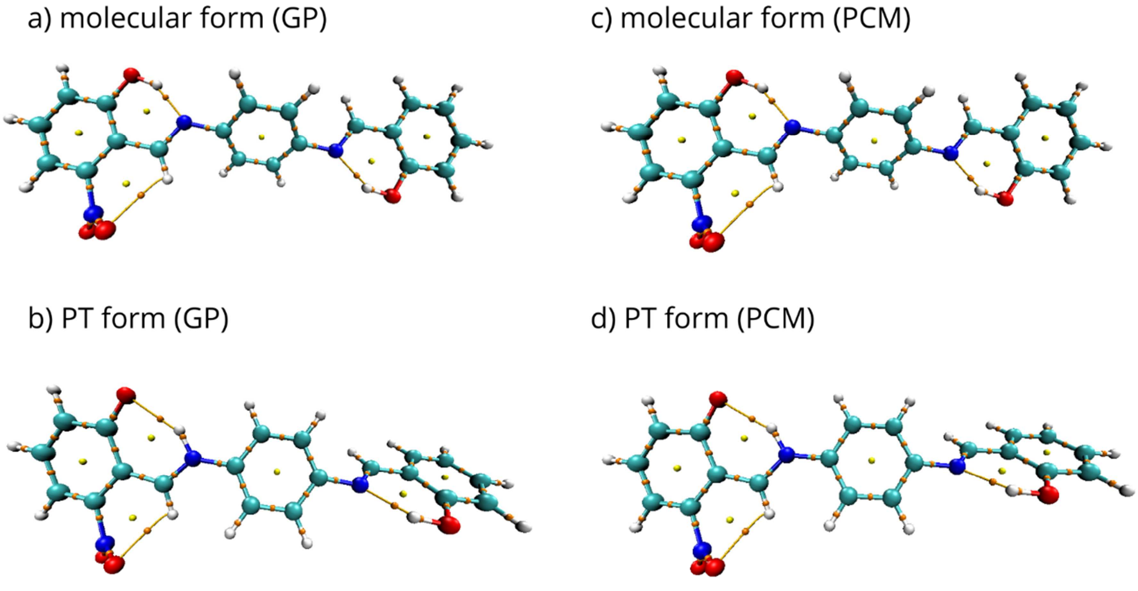

Figure SC5. QTAIM topology maps for para the isomer monosubstituted with the NO_2 group in the proximal position 4: a) molecular form in the gas phase, b) proton-transferred form in the gas phase, c) molecular form in PCM, d) proton-transferred form in PCM. The orange and yellow dots mark the BCPs and RCPs, respectively. Yellow lines pinpoint the intramolecular interaction paths.

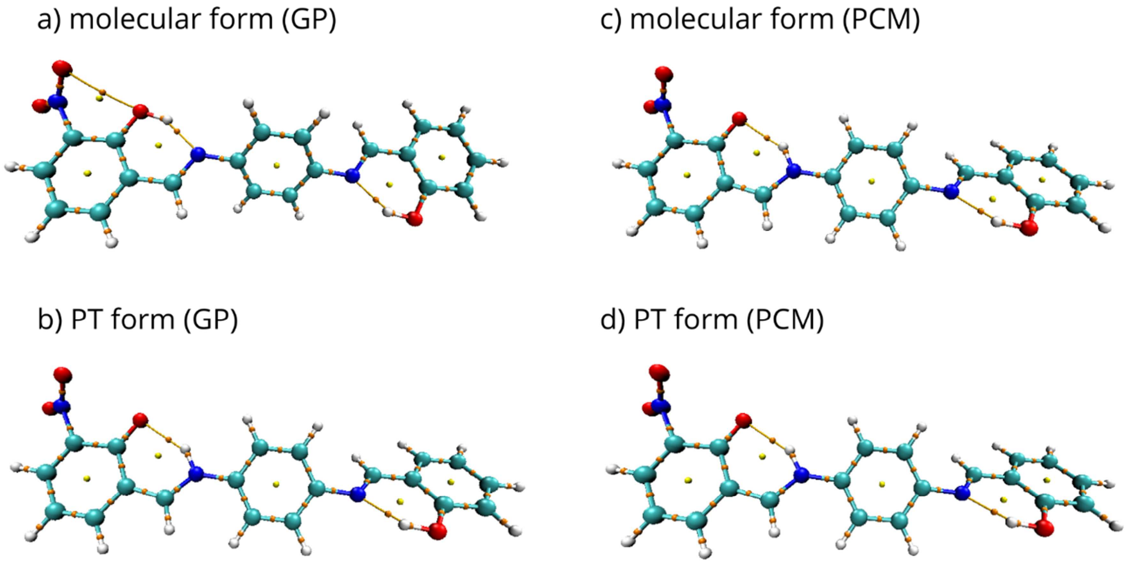

Figure SC6. QTAIM topology maps for the para isomer monosubstituted with the Br atom in the proximal position 1: a) molecular form in the gas phase, b) proton-transferred form in the gas phase, c) molecular form in PCM, d) proton-transferred form in PCM. The orange and yellow dots mark the BCPs and RCPs, respectively. Yellow lines pinpoint the intramolecular interaction paths.

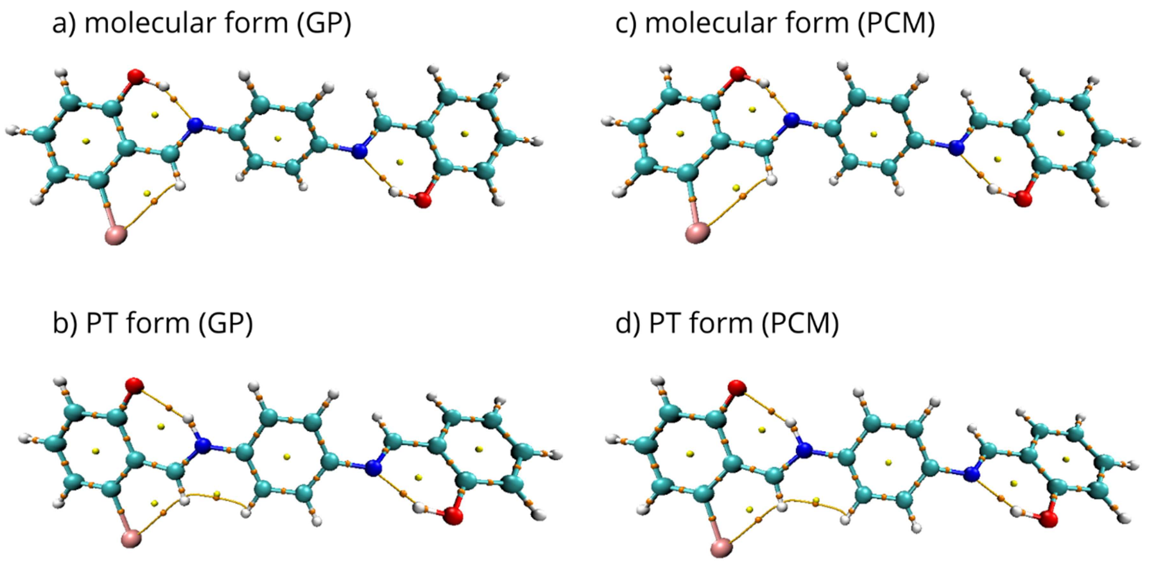

Figure SC7. QTAIM topology maps for the para isomer monosubstituted with the Br atom in the proximal position 4: a) molecular form in the gas phase, b) proton-transferred form in the gas phase, c) molecular form in PCM, d) proton-transferred form in PCM. The orange and yellow dots mark the BCPs and RCPs, respectively. Yellow lines pinpoint the intramolecular interaction paths.

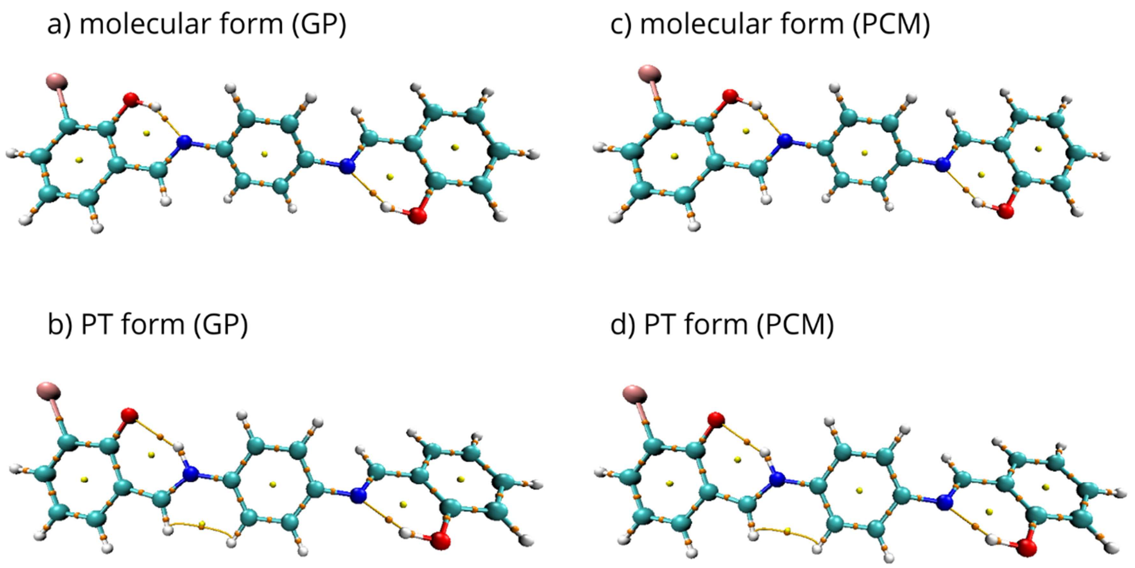

Figure SC8. NCI gradient isosurfaces and scatter graphs for the nonsubstituted para isomer in the gas phase: a) molecular form, b) proton-transferred form.

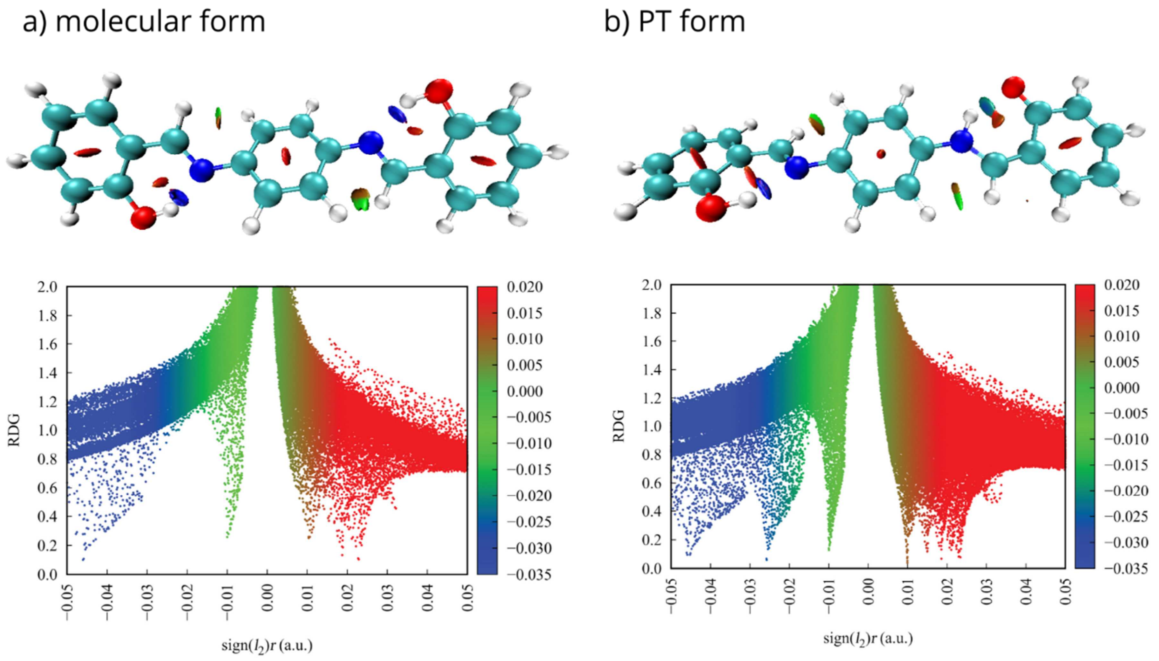

Figure SC9. NCI gradient isosurfaces and scatter graphs for the para isomer monosubstituted with the NH_2 group in the proximal position 1 in the gas phase: a) molecular form, b) proton-transferred form.

a) molecular form

b) PT form

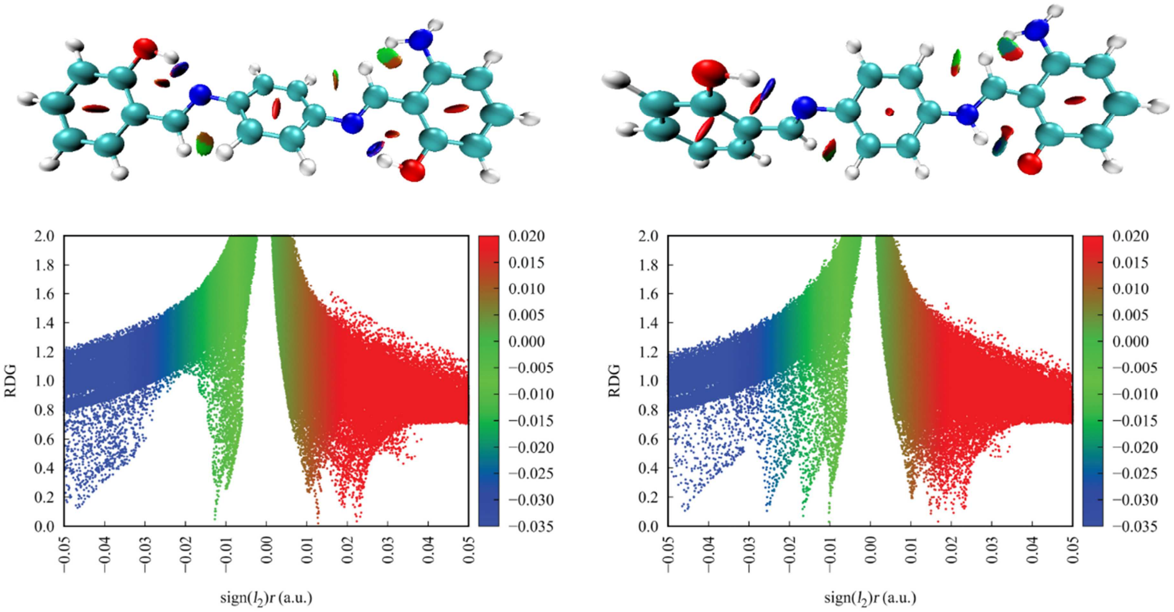

Figure SC10. NCI gradient isosurfaces and scatter graphs for the para isomer monosubstituted with the NH_2 group in the proximal position 4 in the gas phase: a) molecular form, b) proton-transferred form.

a) molecular form

b) PT form

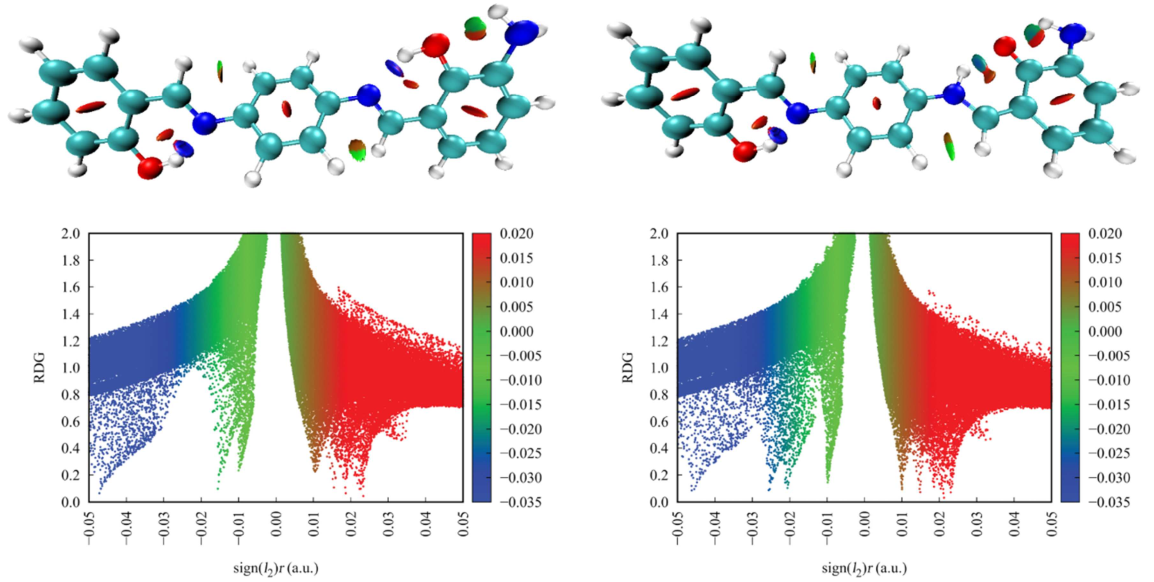

Figure SC11. NCI gradient isosurfaces and scatter graphs for the para isomer monosubstituted with the NO_2 group in the proximal position 1 in the gas phase: a) molecular form, b) proton-transferred form.

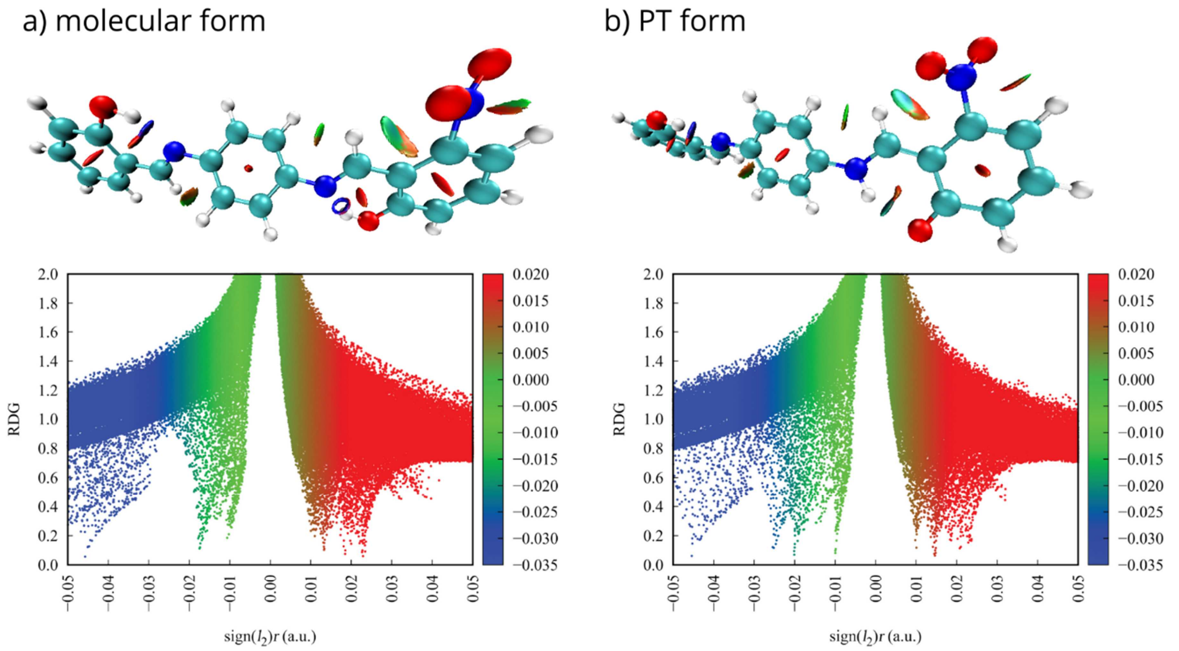

Figure SC12. NCI gradient isosurfaces and scatter graphs for the para isomer monosubstituted with the NO_2 group in the proximal position 4 in the gas phase: a) molecular form, b) proton-transferred form.

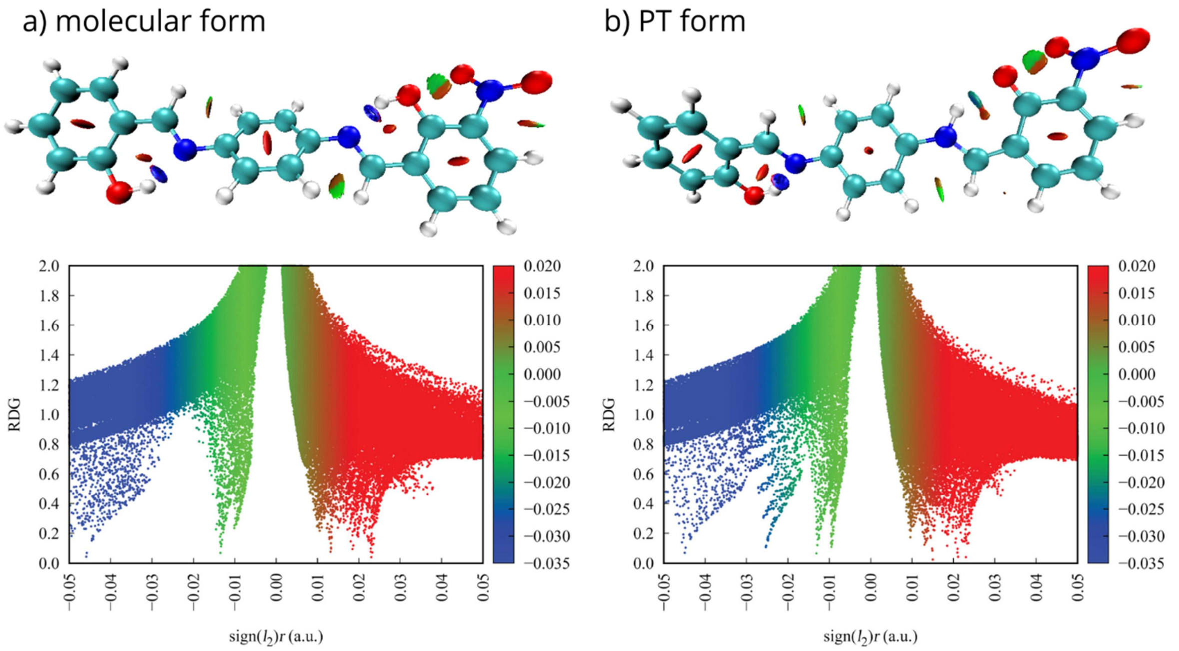

Figure SC13. NCI gradient isosurfaces and scatter graphs for the para isomer monosubstituted with the Br atom in the proximal position 1 in the gas phase: a) molecular form, b) proton-transferred form.

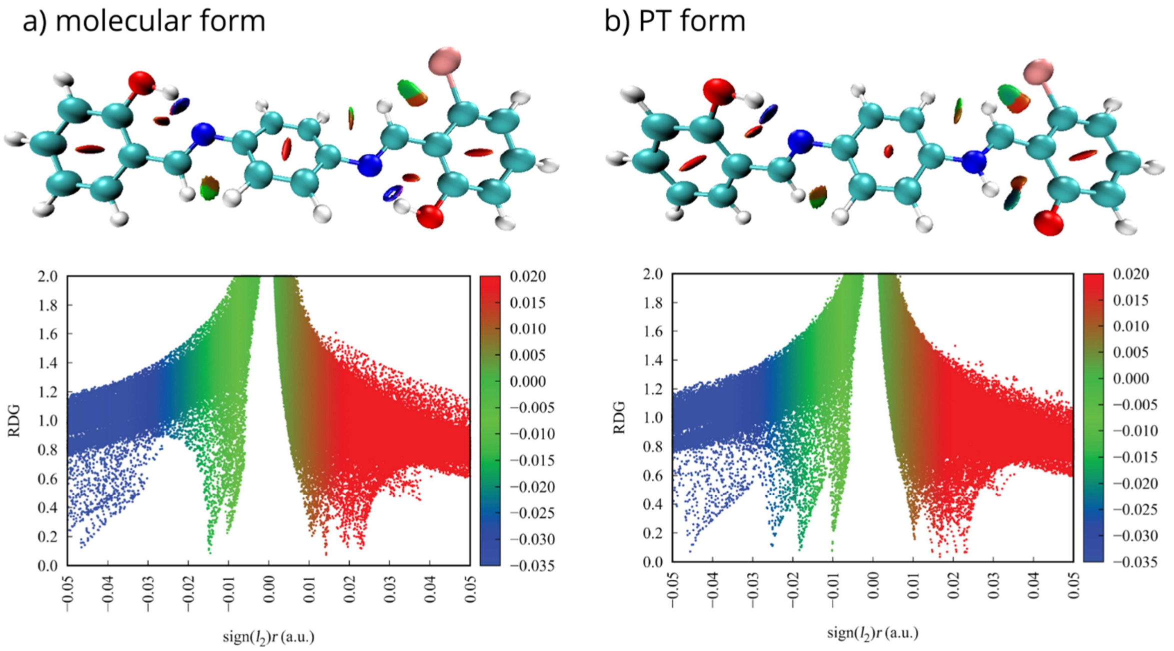

Figure SC14. NCI gradient isosurfaces and scatter graphs for the para isomer monosubstituted with the Br atom in the proximal position 4 in the gas phase: a) molecular form, b) proton-transferred form.

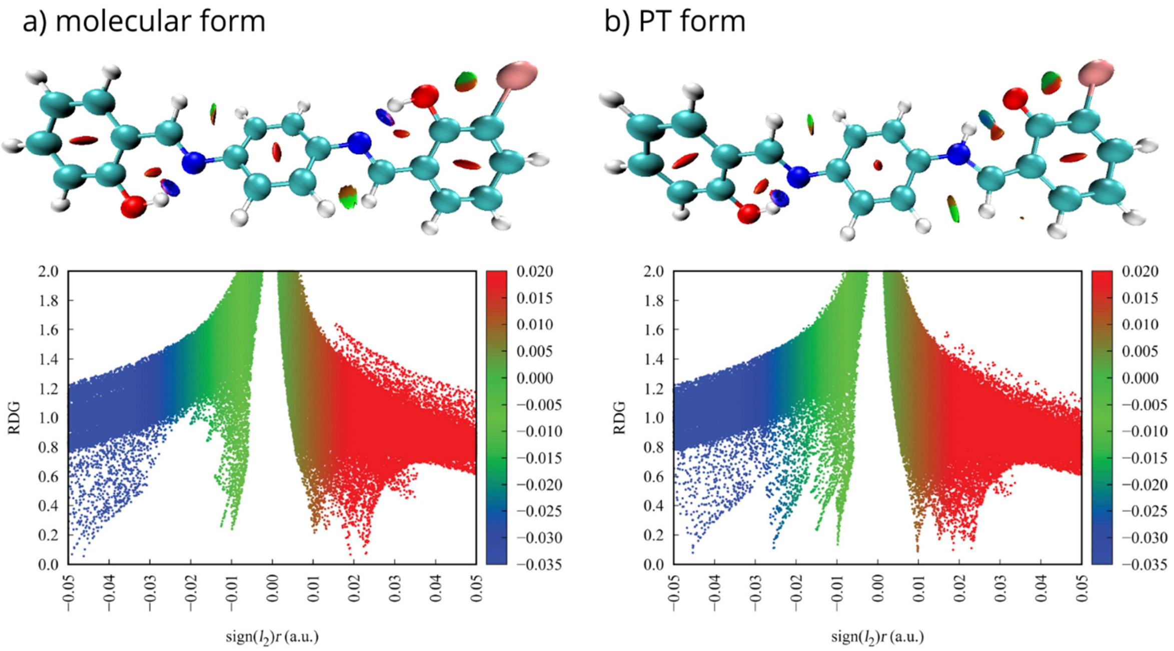

Figure SC15. NCI gradient isosurfaces and scatter graphs for the nonsubstituted para isomer in PCM: a) molecular form, b) proton-transferred form.

a) molecular form

b) PT form

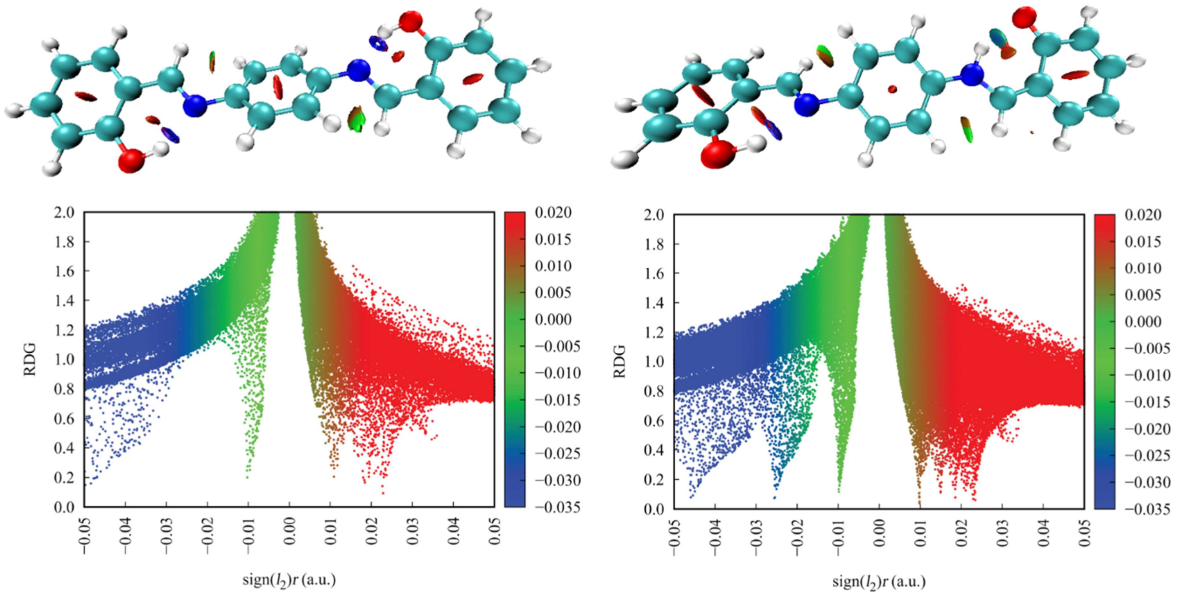

Figure SC16. NCI gradient isosurfaces and scatter graphs for the para isomer monosubstituted with the NH_2 group in the proximal position 1 in PCM: a) molecular form, b) proton-transferred form.

a) molecular form

b) PT form

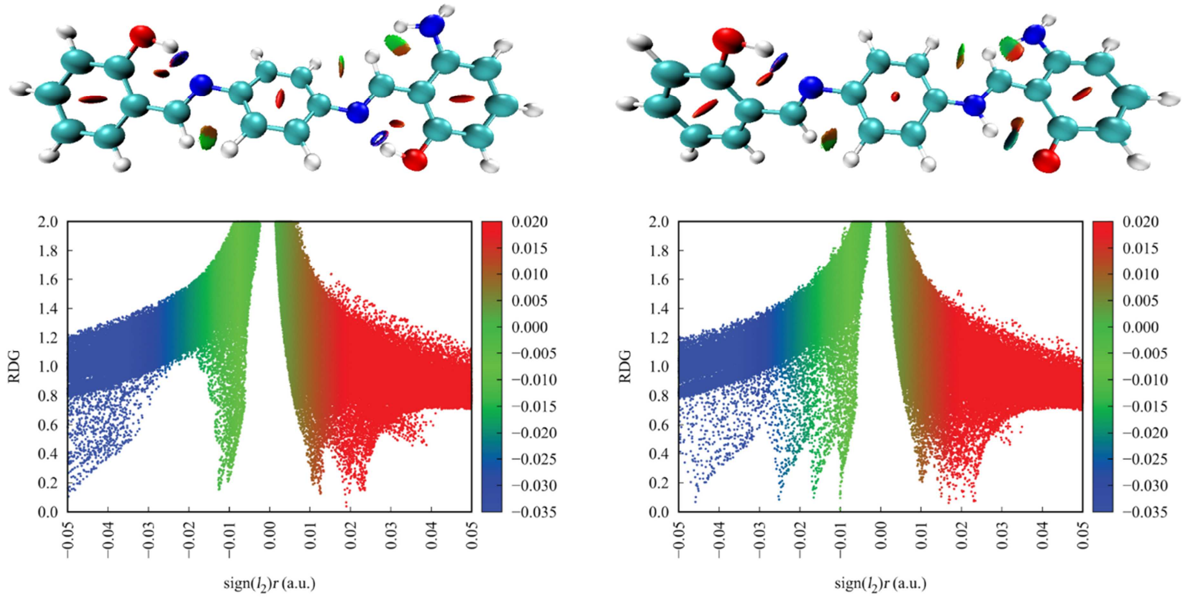

Figure SC17. NCI gradient isosurfaces and scatter graphs for the para isomer monosubstituted with the NH_2 group in the proximal position 4 in PCM: a) molecular form, b) proton-transferred form.

a) molecular form b) PT form

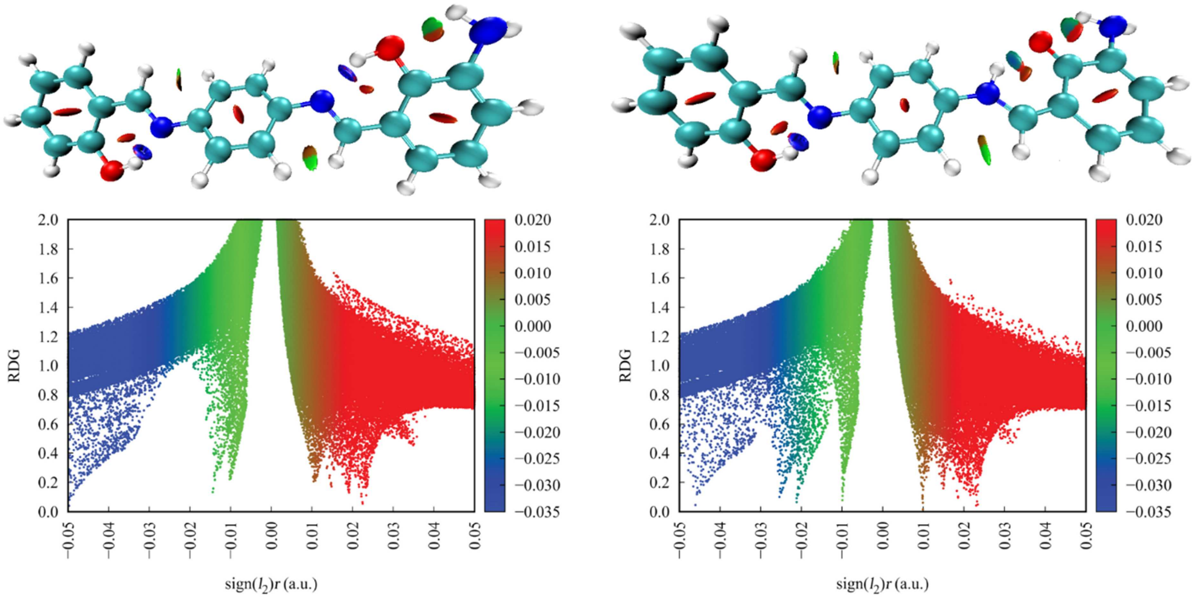

Figure SC18. NCI gradient isosurfaces and scatter graphs for the para isomer monosubstituted with the NO_2 group in the proximal position 1 in PCM: a) molecular form, b) proton-transferred form.

a) molecular form b) PT form

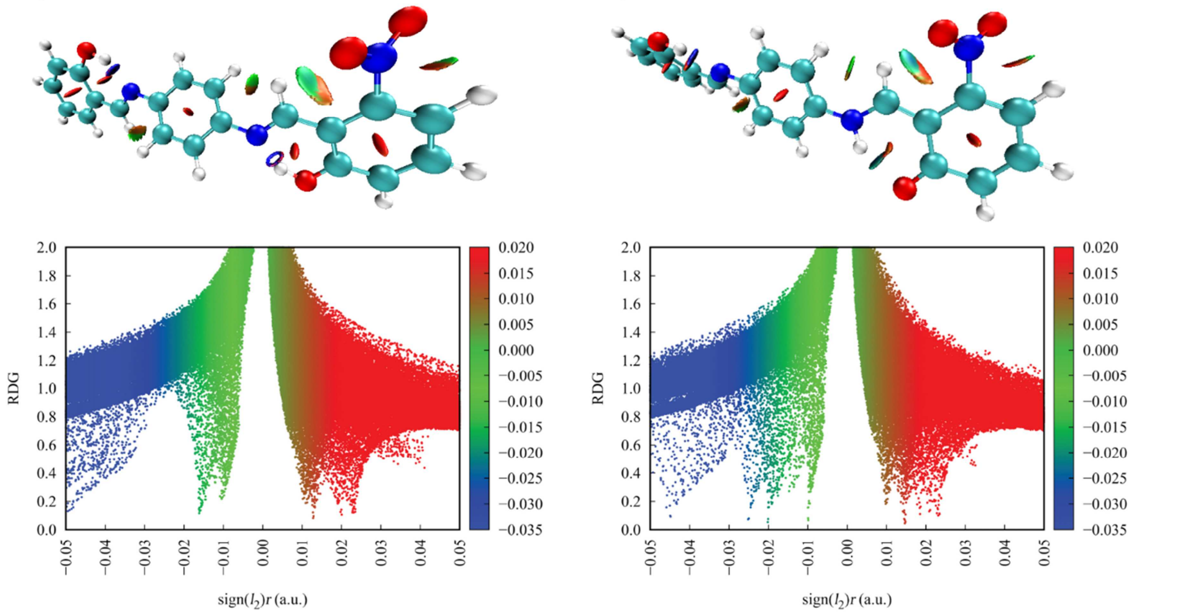

Figure SC19. NCI gradient isosurfaces and scatter graphs for the para isomer monosubstituted with the NO_2 group in the proximal position 4 in PCM: a) molecular form, b) proton-transferred form.

a) molecular form b) PT form

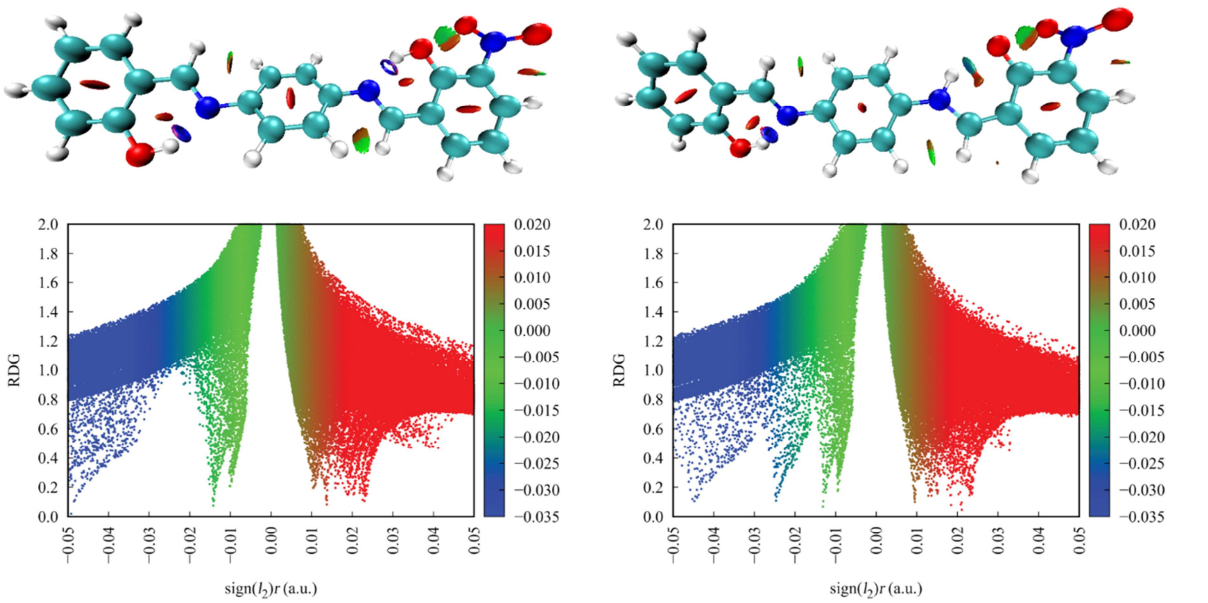

Figure SC20. NCI gradient isosurfaces and scatter graphs for the para isomer monosubstituted with the Br atom in the proximal position 1 in PCM: a) molecular form, b) proton-transferred form.

a) molecular form b) PT form

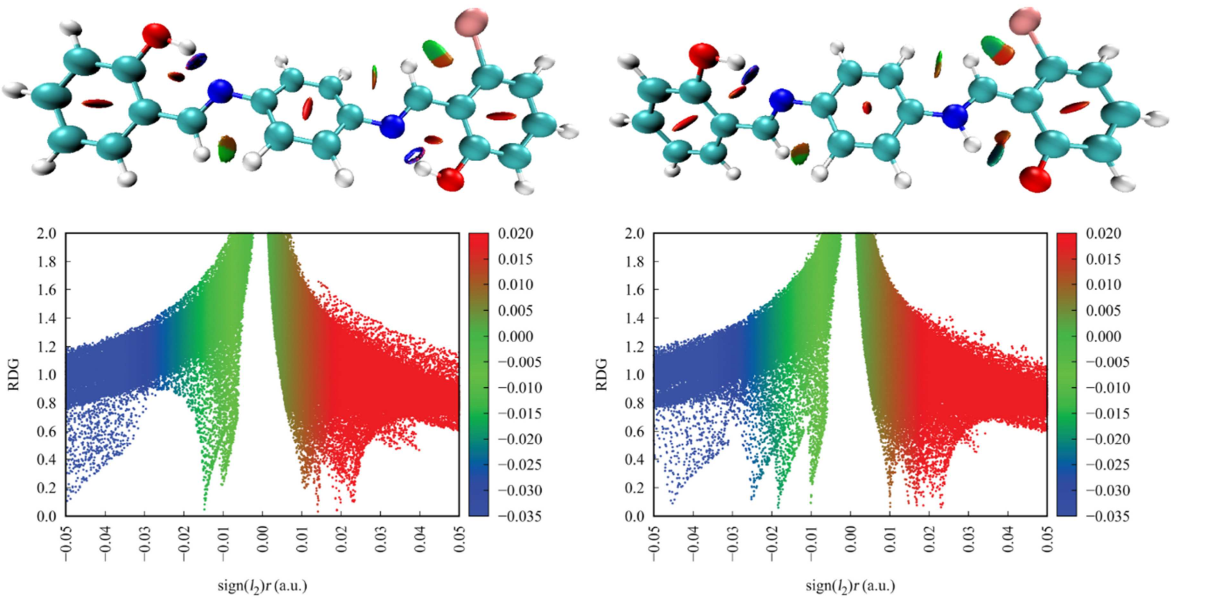

Figure SC21. NCI gradient isosurfaces and scatter graphs for the para isomer monosubstituted with the Br atom in the proximal position 4 in PCM: a) molecular form, b) proton-transferred form.

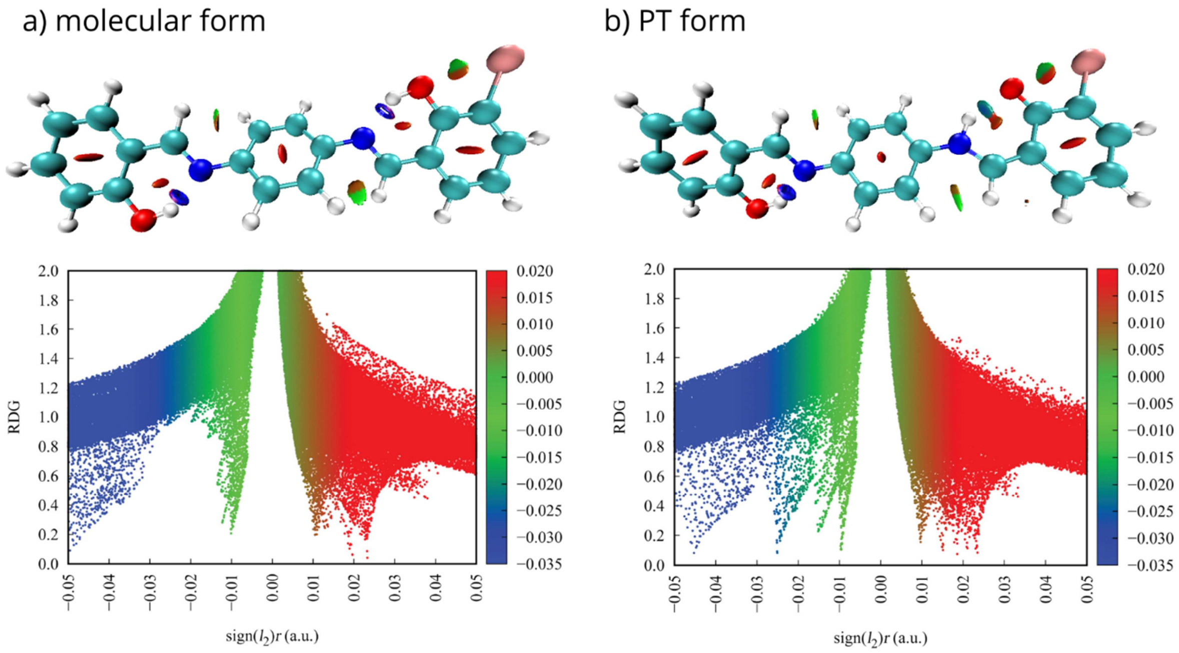

Supplement: Supplementary file 1 [file ijms-23-12439-s001.zip › Supplement C.pdf]
